# Supplementary material for: Continuous and low-carbon production of biomass flash graphene
Source: Nat Commun. 2024 Apr 15;15:3218. doi: 10.1038/s41467-024-47603-y (PMC11018853; doi:10.1038/s41467-024-47603-y)
Supplement: Supplementary file 1 — Supplementary Information [file 41467_2024_47603_MOESM1_ESM.pdf]

Supplementary information

Continuous and low-carbon production of biomass flash graphene

Xiangdong Zhu,*[1,2,§] Litao Lin,[1,3,§] Mingyue Pang,[4,§] Chao Jia,[1] Longlong Xia,[2] Guosheng Shi,[5] Shicheng Zhang,[1] Yuanda Lu,[1] Liming Sun,[1] Fengbo Yu,[1] Jie Gao,[1] Zhelin He,[1] Xuan Wu,[1] Aodi Li,[1] Liang Wang,[3] Meiling Wang,[6] Kai Cao,[6] Weiguo Fu,[6] Huakui Chen,[6] Gang Li,[7] Jiabao Zhang,[2] Yujun Wang,*[2] Yi Yang,*[4] and Yong-Guan Zhu,*[8,9]

[1] *Department of Environmental Science and Engineering, Fudan University, Shanghai 200433, China.*

[2] *State Key Laboratory of Soil and Sustainable Agriculture, Institute of Soil Science, Chinese Academy of Sciences, Nanjing 210018, China.*

[3] *School of Energy and Power, Jiangsu University of Science and Technology, Zhenjiang, Jiangsu 212003, China.*

[4] *Key Laboratory of Three Gorges Reservoir Region's Eco-Environment, Ministry of Education, Chongqing University, Chongqing 400044, China.*

[5] *Shanghai Applied Radiation Institute and State Key Laboratory Advanced Special Steel, Shanghai University, Shanghai 200444, China.*

[6] *Institute of Intelligent Machines Hefei Institutes of Physical Science, Chinese Academy of Sciences, Changzhou 213164, China.*

[7] *Key Lab of Urban Environment and Health, Institute of Urban Environment, Chinese Academy of Sciences, Xiamen 361021, China.*

[8] *State Key Laboratory of Urban and Regional Ecology, Research Center for Eco-Environmental Sciences, Chinese Academy of Sciences, Beijing 100085, China.*

[9] *Zhejiang Key Laboratory of Urban Environmental Processes and Pollution Control, CAS Haixi Industrial Technology Innovation Center in Beilun, Ningbo 315830, China.*

** Corresponding author E-mail:*

zxdjewett@fudan.edu.cn *(Xiangdong Zhu);*

yjwang@issas.ac.cn (*Yujun Wang*);
yi.yang@cqu.edu.cn (Yi Yang);
ygzhu@rcees.ac.cn (Yong-Guan Zhu).
§ These three authors contributed equally to this paper.

Contents:

Supplementary Materials and Methods

Supplementary Figures from 1 to 47

Supplementary Tables from 1 to 19

Supplementary References

Supplementary note: precautions and instructions of integrated device

1. Precautions

1.1 Guidance for operation personnel

Learning operational flow, troubleshooting, consumables replacement, security protection precautions and device cleaning (reactive electrode and sample sink).

1.2 Power supply system management

(1) Ensure the device is grounded before operating.

(2) Ensure insulation between the body and the system.

(3) Turn off the power and pull off the circuit breaker after production.

1.3 Prevention of safety accidents

(1) Avoid the involvement of other staff around the production site. Do not interfere with the operation of the device when it is put into operation.

(2) Committed to carefully inspecting the engineering site to prevent accidents (such as fire hazard), especially high-voltage FJH discharge components.

(3) Inflammable materials shall not be placed on the production site.

(4) In the possibility event of emergency, expeditiously stop the device operation.

(5) Press the emergency stop button or turn off the power during device inspection.

2 Instructions

2.1 Operation Panel

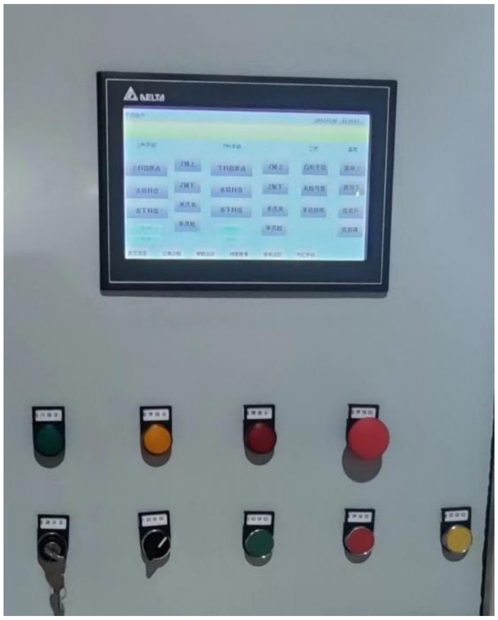

Supplementary Fig. 1 | Photograph of operation panel. The following text describes the functions of the button and indicator:

"Running indicator": System is in automatic operation mode, and the running indicator light is on.

"Pause Indicator": During the automatic operation of the system, after pressing the pause button, the system enters the pause mode, and the pause indicator lights up.

"Alarm indicator": The alarm light will be lit when the system has a fault alarm.

"Power switch": The lamp is lit when the power load switch is turned on. Currently, the controller and servo are not powered on. The user powers on the control system through the "power switch".

"Manual automatic": The manual/automatic switch selects the operation mode of the

system and turns to "manual", and the system works in the manual state; Turn to "automatic", the system working mode is switched to automatic after startup, and the manual function is invalid.

"Start Button": The system runs automatically. When ready, press the start button, and the system runs automatically.

"Pause Button": The system starts automatically. After pressing the pause button, the system stops running.

"Emergency stop button": Pressed in case of mis-operation or emergency, the system will immediately stop; To prevent accidents, the button is self-locking. After removing the fault, the button can be rotated to release the self-locking state. After pressing the "continue running" button, the system enters the automatic running state again.

2.2 Device operation

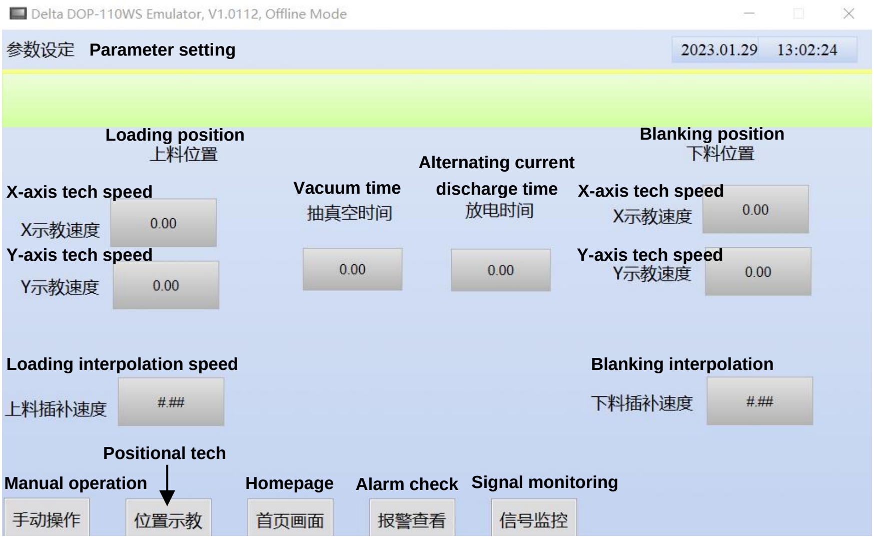

Supplementary Fig. 2 | Parameter setting page. This page mainly provides the setting of the vacuum time, alternating current discharge time, biaxial teaching velocity, and

interpolation motion velocity.

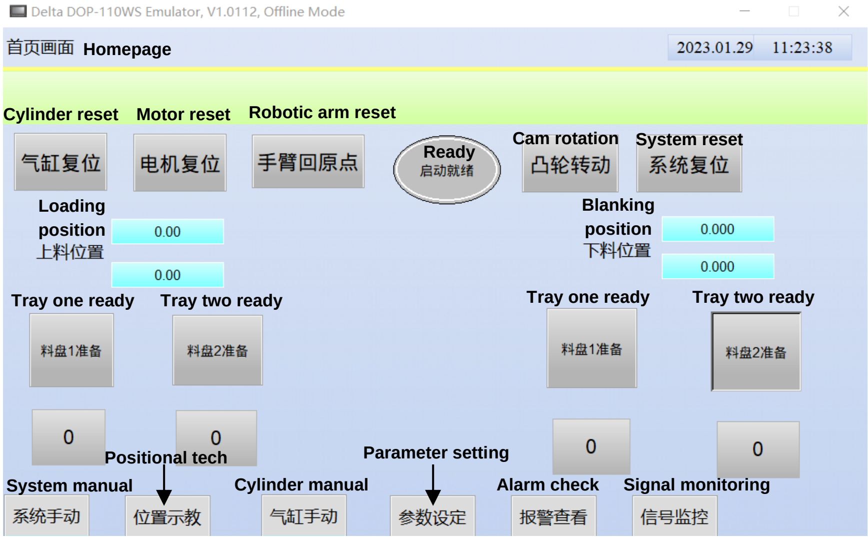

Supplementary Fig. 3 | Fabrication and setting of blanking tray of continuous production process.

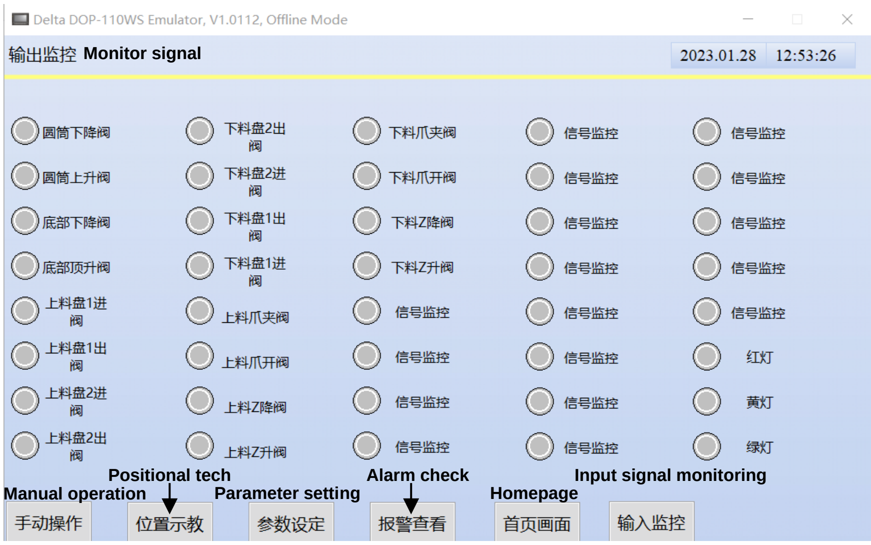

Supplementary Fig. 4 | Monitor signal page of continuous production process.

Supplementary Table 1. Express the text in the above output monitor signal page in English.

First column	Second column	Third column	Fourth column	Fifth column
Cylinder drop valve	Blanking tray one enter valve	Blanking claw clamp valve	Signal monitoring	Signal monitoring
Cylinder rise valve	Blanking tray one exit valve	Blanking claw release valve	Signal monitoring	Signal monitoring
Bottom electrode drop valve	Blanking tray two enter valve	Z-axis Blanking drop valve	Signal monitoring	Signal monitoring
Bottom electrode rise valve	Blanking tray two exit valve	Z-axis Blanking rise valve	Signal monitoring	Signal monitoring
Loading tray one enter valve	Loading claw clamp valve	Signal monitoring	Signal monitoring	Signal monitoring
Loading tray one exit valve	Loading claw release valve	Signal monitoring	Signal monitoring	Red light
Loading tray two enter valve	Z-axis Loading drop valve	Signal monitoring	Signal monitoring	Yellow light
Loading tray two exit valve	Z-axis Loading rise valve	Signal monitoring	Signal monitoring	Green light

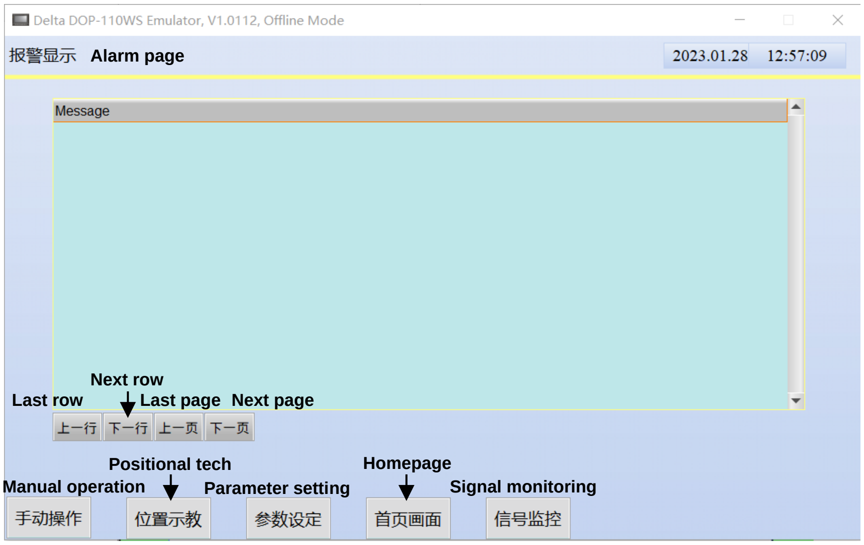

Supplementary Fig. 5 | Display alarm information of operation system on alarm page.

Supplementary Table 2 | Component lists of continuous integrated production device and their corresponding functions. The capacitors used in lab-scale flash graphene production device is 63.8 mF, while in pilot-scale fabrication device is 127.6 mF.

**Component**	**Function**
Human-machine interfaces	System parameter setting
Vacuum pumps	Vacuum extraction
Air compressor<br>Servo motors<br>Pneumatic solenoid valves<br>Servo controller	Power supply system
Cam indexer<br>Truss manipulator	Loading and blanking
Intermediate relay<br>AC-DC converter<br>PLC Controller<br>Input expansion module of PLC controller<br>Output expansion module of PLC controller	Electrical control system

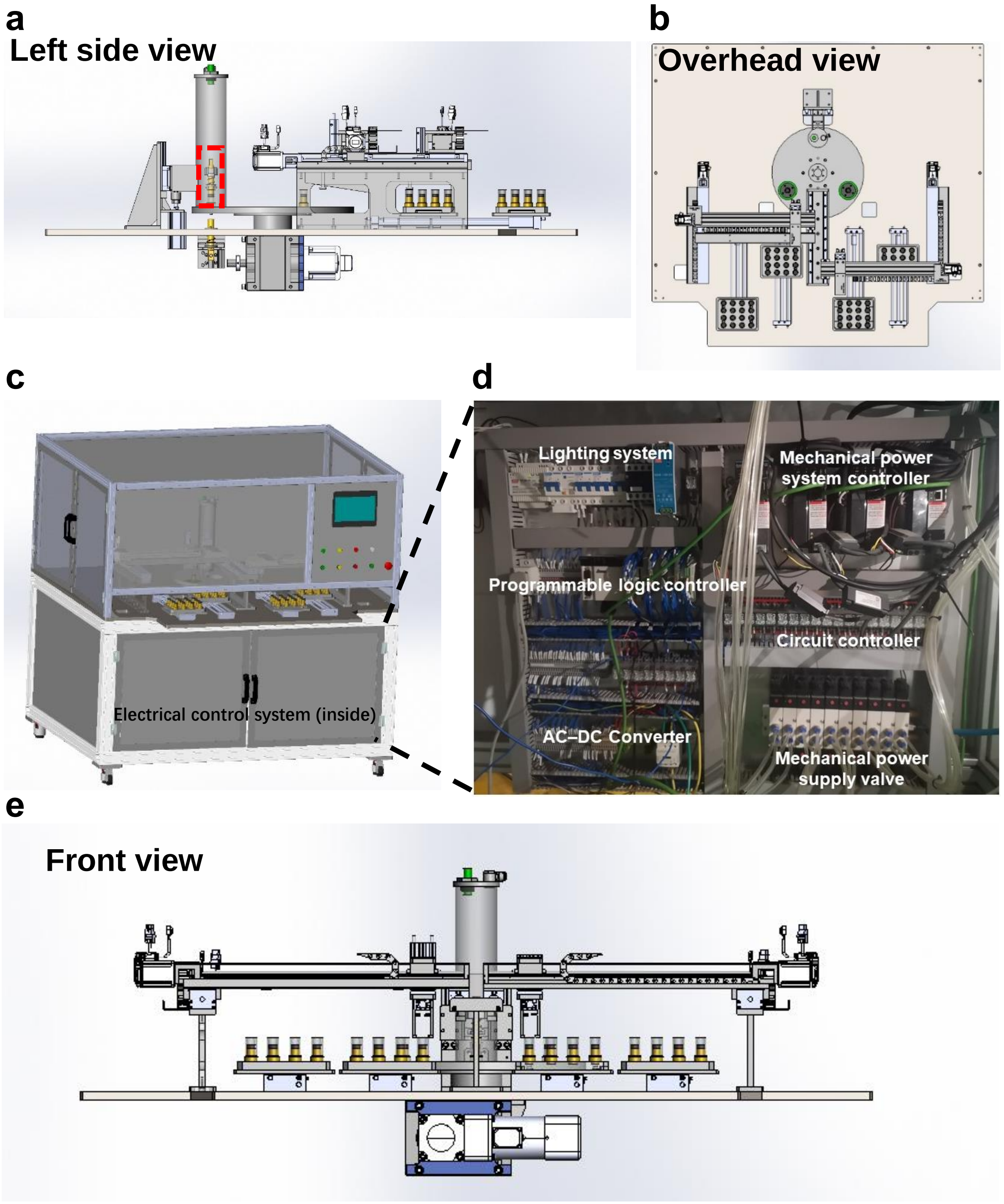

Supplementary Fig. 6 | Design and interior diagram of integrated device. a, Left side view of device. **b,** Overhead view of device. In the red dotted box, because the volume of the packed sample in the quartz tube could be slightly different, there may be insufficiently pressed between the samples and reaction electrodes, resulting in an air gap affecting

the biomass FG fabrication. Therefore, we design a flexible compress device with pre-compaction to ensure that each sample is fully compressed with the electrode, thus reducing the biomass FG structural differences caused by the inconsistent pressing force. **c**, Overall view of device. **d**, Interior electrical control system. **e**, Front view of device.

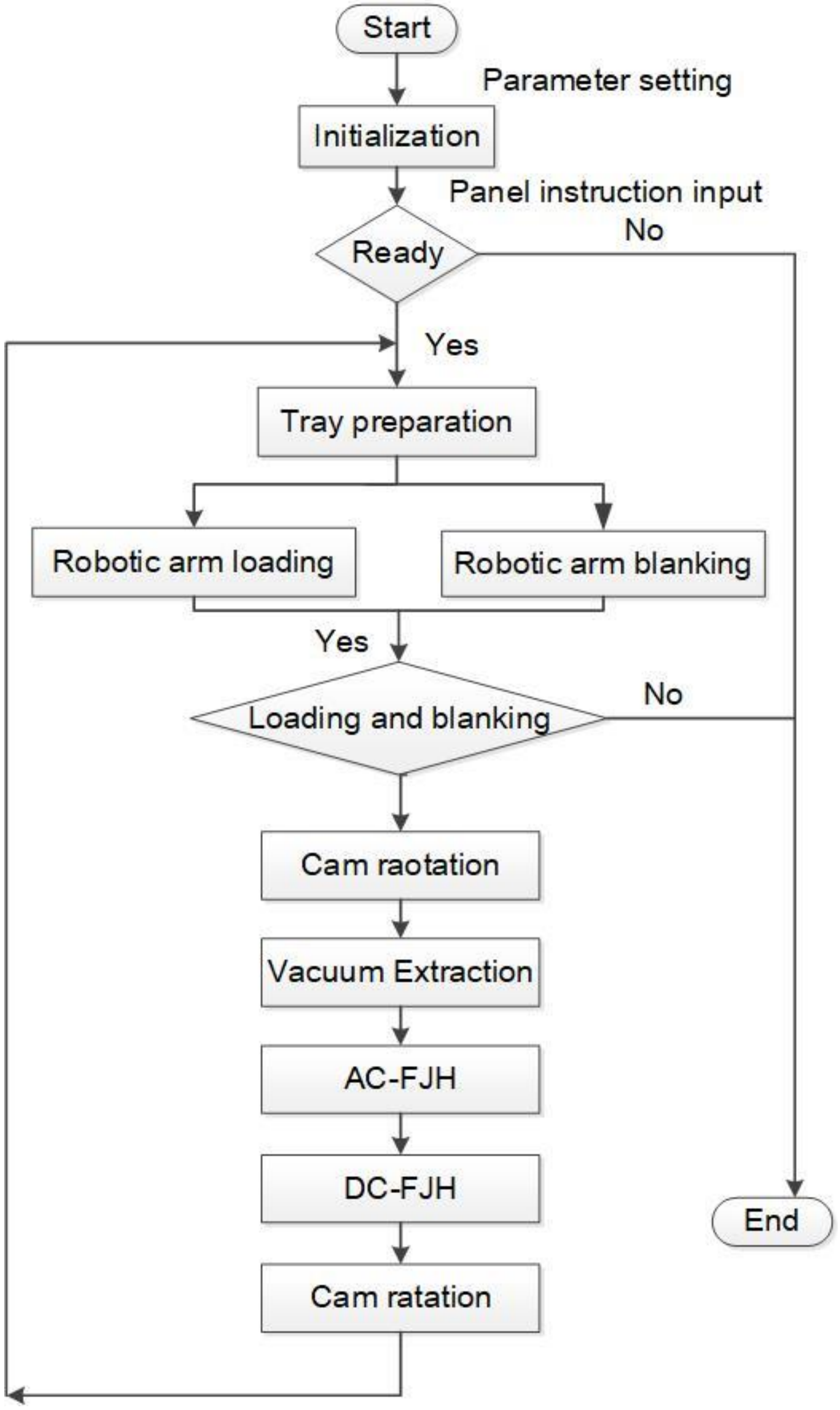

Supplementary Fig. 7 | Logical framework for continuous production system at pilot-scale. Step switching is conditional (such as time), and only the conditions that are met can proceed to the next step. "Robotic arm blanking" means that a robotic arm is

used to transport the sample from the loading sample tray to the flash Joule heating reaction area. "Robotic arm blanking" means to grab the sample from the flash Joule heating reaction area to the blanking sample tray.

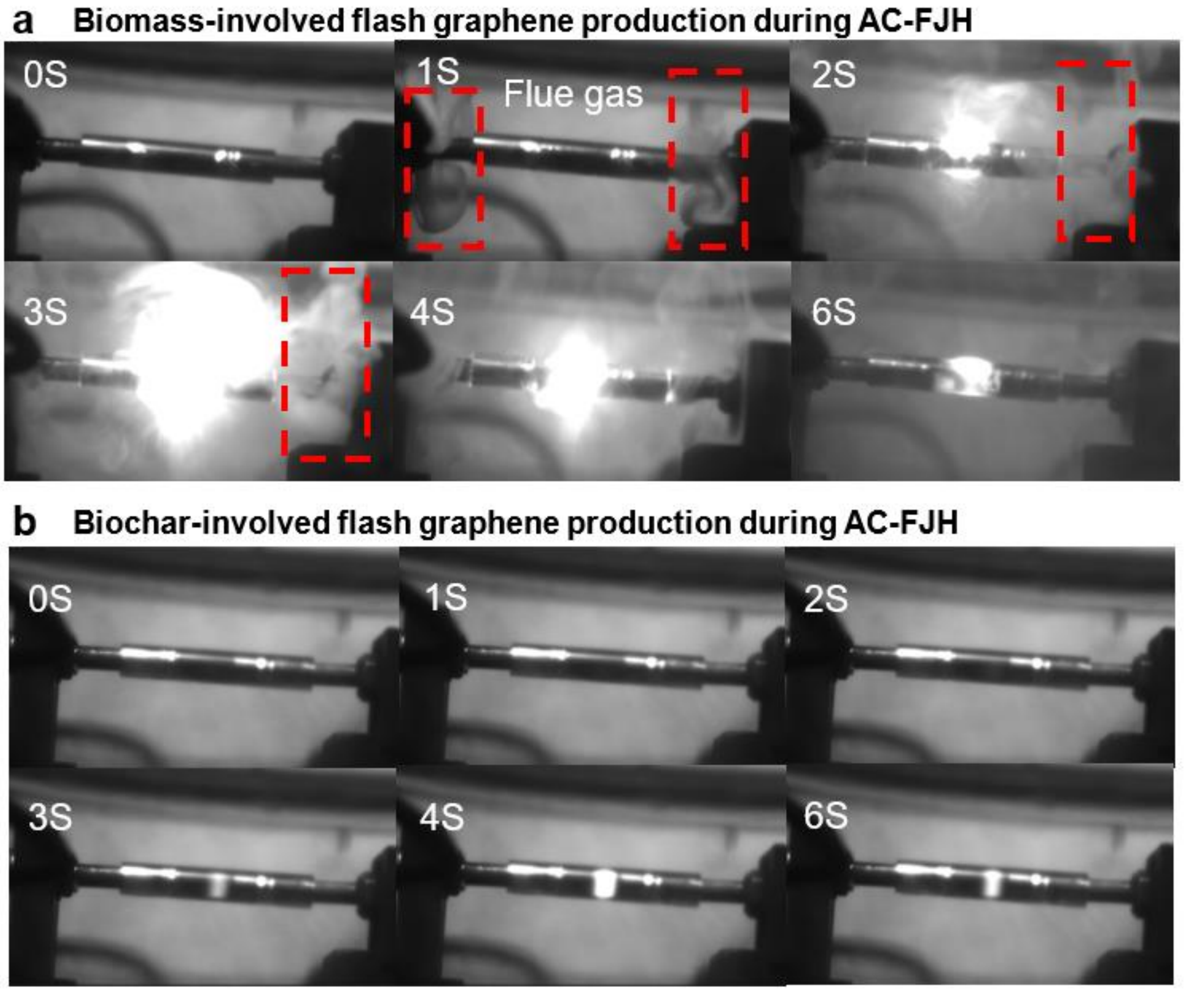

Supplementary Fig. 8 | Digital images of flash graphene production process during AC-FJH. a, Dynamic image record of biomass-based flash graphene production in the AC-FJH process within six seconds. **b**, Dynamic images record of 750 °C biochar-involved flash graphene production in the AC-FJH process.

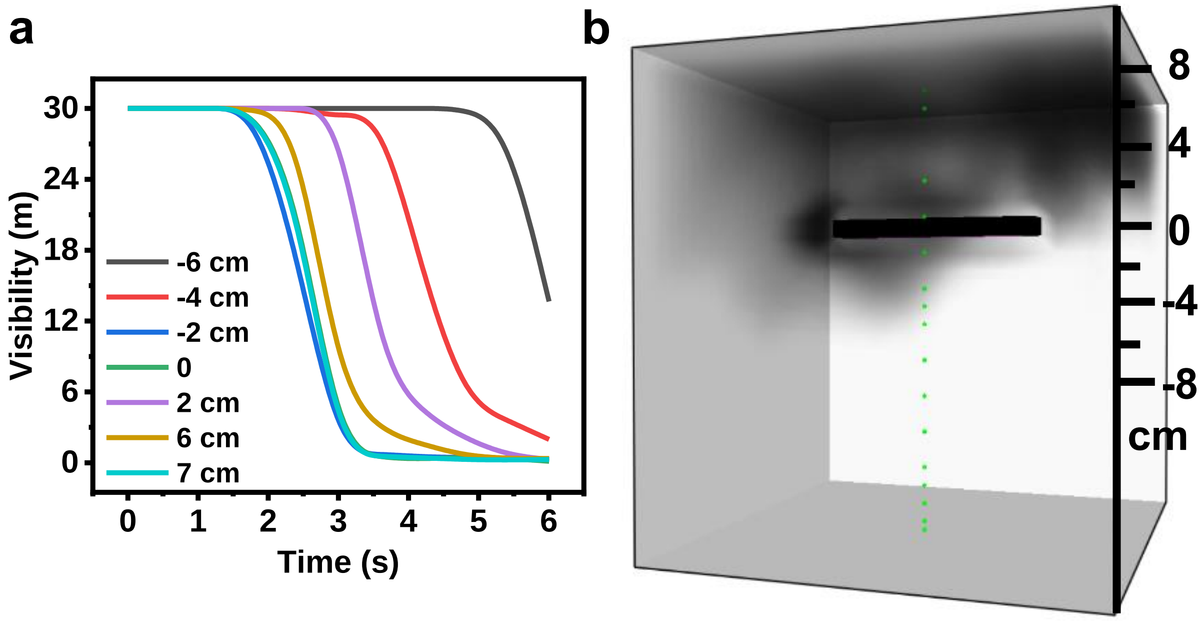

Supplementary Fig. 9 | a, Visibility of biomass-based flash graphene production during alternating current flash Joule heating was simulated by fire dynamics simulator. **b,** Simulation results show that massive unrestrained biomass pyrolytic volatiles are released during AC-FJH process. Fire dynamics simulator is developed by the National Institute of Standards and Technology in the United States, and we use version 6.3.2.

Supplementary Table 3. Gas species generated in biomass (sawdust) and sawdust-derived 300°C biochar-based flash graphene production process during AC-FJH.

Gas species (vol %)	Sawdust	Sawdust-derived 300°C biochar
Hydrogen (H_2)	24.5	21.7
Carbon monoxide (CO)	36.0	34.8
Carbon dioxide (CO_2)	5.56	2.81
Methane (CH_4)	3.64	3.04
Acetylene (C_2H_2)	3.87	5.22
Ethylene (C_2H_4)	2.70	0.66
Ethane (C_2H_6)	0.52	0.27
Propylene (C_3H_6)	0.80	0.25
Propane (C_3H_8)	0.05	0.05
Other	22.4	31.2

To figure out the gas composition, the collected bio-gas compositions were quantitatively analyzed by gas chromatography (GC-960, Haixin, China) equipped with a thermal conductivity detector channel. To detect hydrogen, the carrier gas was nitrogen and the temperatures of the injector, detector, and oven were set at 393K, 383K, and 393K, respectively. While examining CO, CO_2, and CH_4, the carrier gas was changed to helium, and other conditions were the same. Hydrocarbons (including C_2H_2, C_2H_4, C_2H_6, C_3H_6, and C_3H_8) were examined on a flame-ionization detector with nitrogen as the carrier gas with the oven temperature of 393K. A standard gas mixture of these hydrocarbons was purchased from Air Liquide Compressed Gas Co., Ltd (China). The response factor was obtained using standard gases for quantitative analysis.

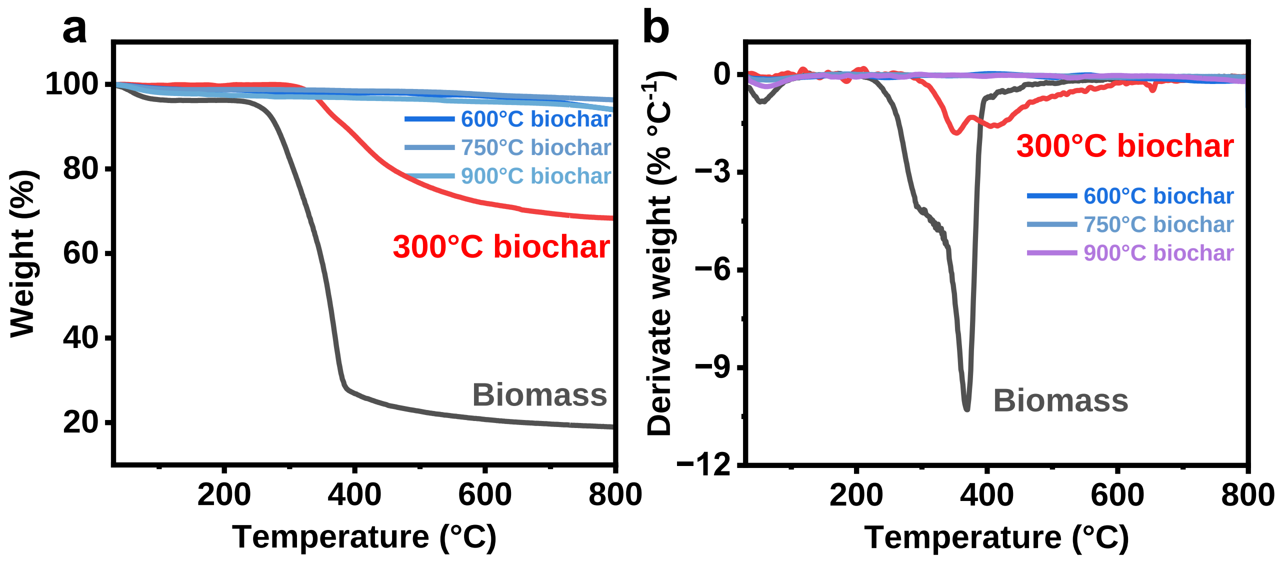

Supplementary Fig. 10 | a, Thermogravimetric and **b,** Derivative Thermogravimetric analysis for biomass (sawdust), and sawdust derived biochar under different carbonization temperature (300, 600, 750, and 900 °C) in the air. Thermal stability of sample was increased with the increase of carbonization temperature.

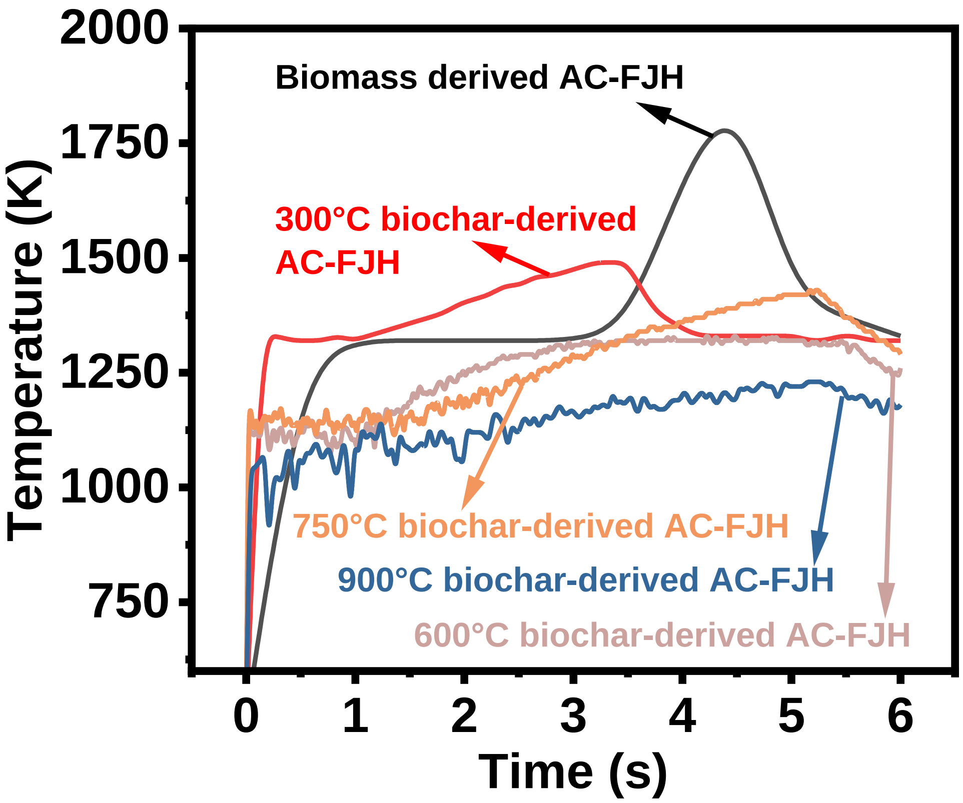

Supplementary Fig. 11 | The real-time reaction temperature of flash graphene production during AC-FJH from various production paths was recorded for six seconds. During the AC-FJH process, the black body radiation from the sample was collected by the optical fiber, and the spectral radiation was recorded by an infrared spectrometer (FX2000, Ideaoptics, China) at 500-1100 nm. Before the measurement, the temperature was calibrated with a mercury lamp. Based on Joule's Law, the heat was generated with increased sample resistance, resulting in a higher reaction temperature. Therefore, the biomass-derived AC-FJH reaction process has the highest reaction temperature due to the highest sample resistance.

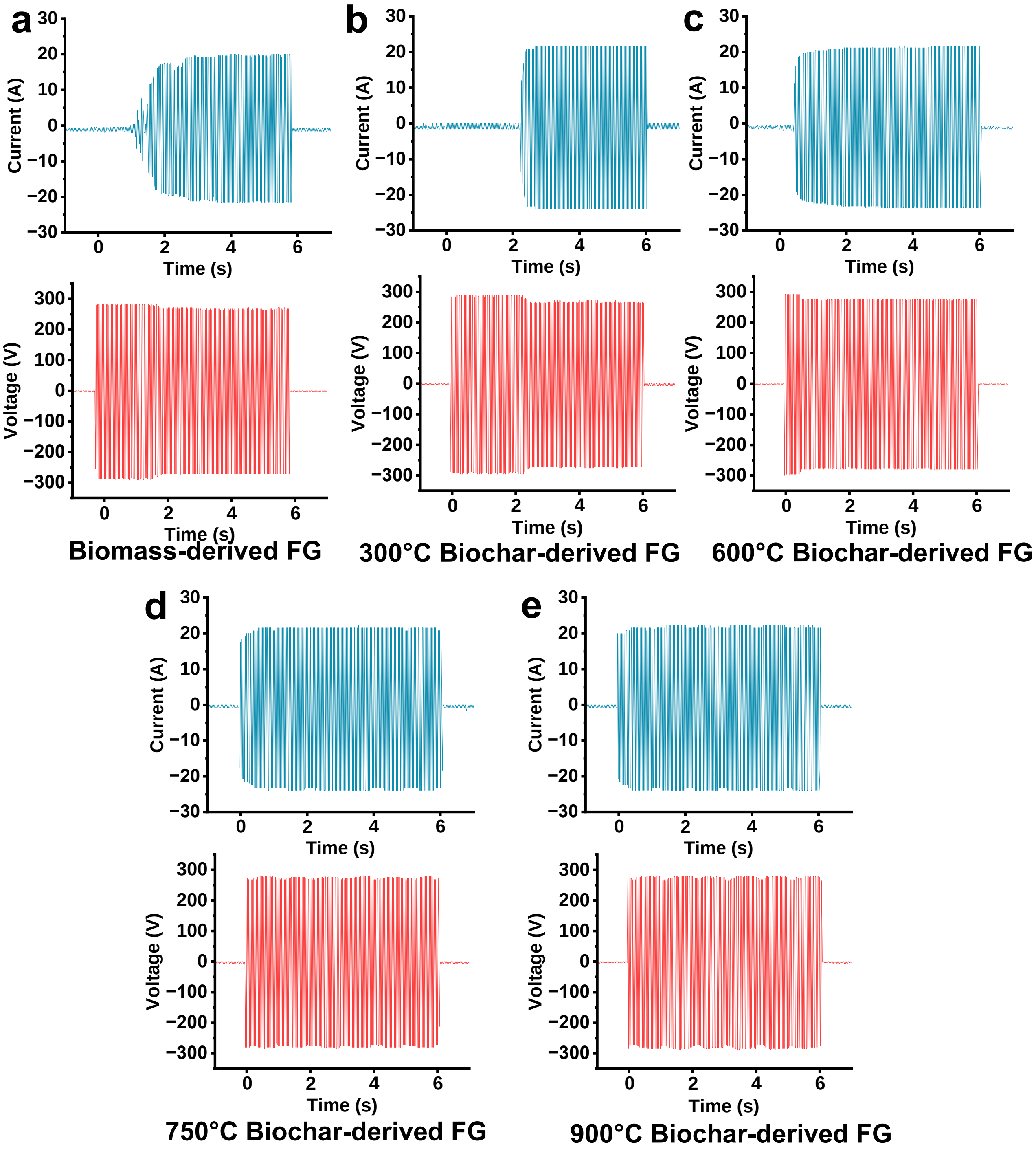

Supplementary Fig. 12 | Current and voltage of flash graphene production paths from **a,** biomass, **b,** 300°C biochar, **c,** 600°C biochar, **d,** 750°C biochar, **e,** 900°C biochar was recorded during AC-FJH for six seconds.

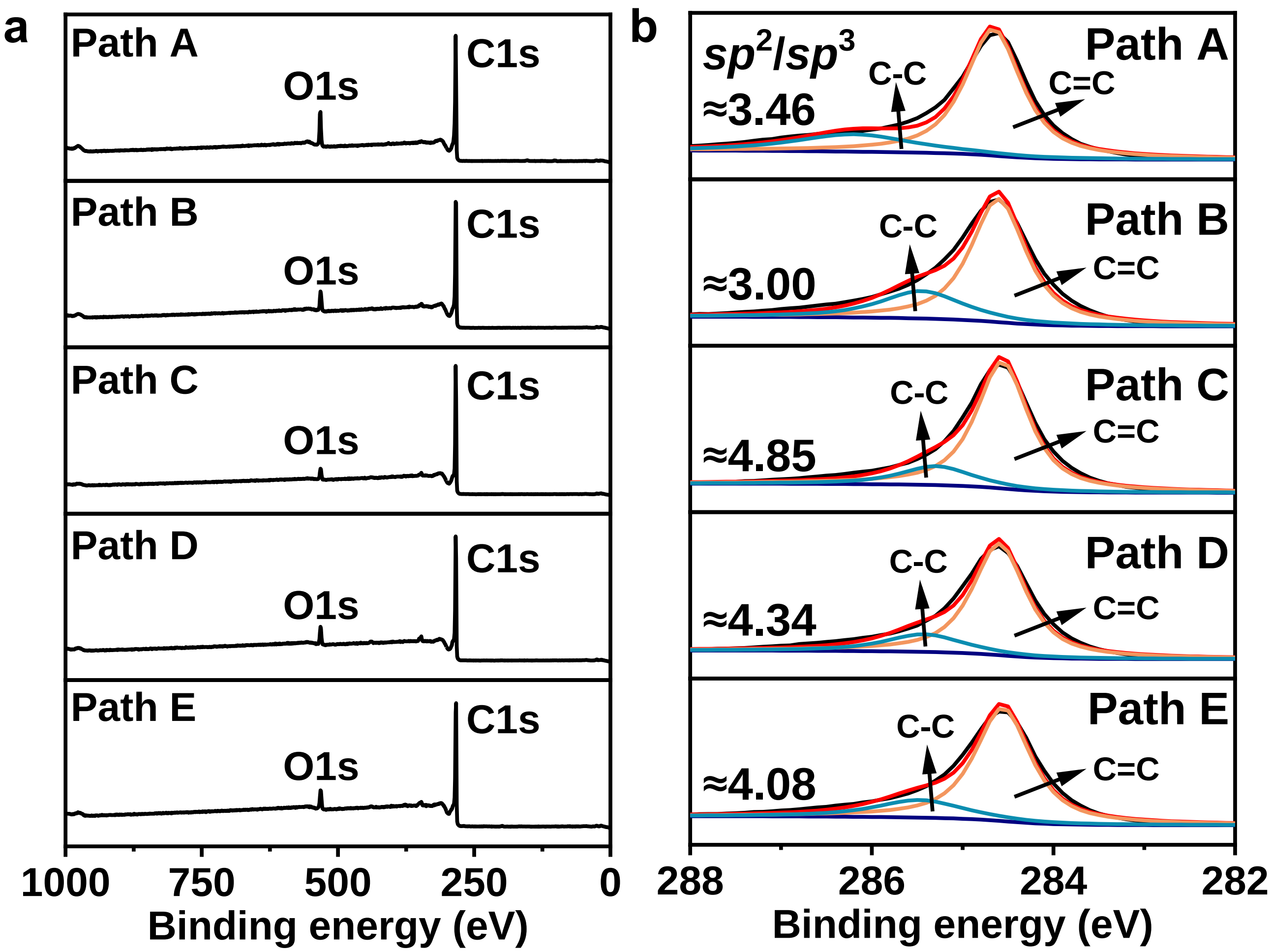

Supplementary Fig. 13 | a, XPS survey scans and **b,** C1 spectra of flash graphene from various production path. Using high-resolution XPS and peak deconvolution to examine the C1 spectra binding region, C=C (*sp*^{2} carbon) and C-C (*sp*^{3} carbon) bond types are detected with peaks at ~284.6 and ~285.3, respectively.

Path A: biomass-based flash graphene production; Path B: 300°C biochar-based flash graphene production; Path C: 600°C biochar-based flash graphene production; Path D: 750°C biochar-based flash graphene production; Path E: 900°C biochar-based flash graphene production.

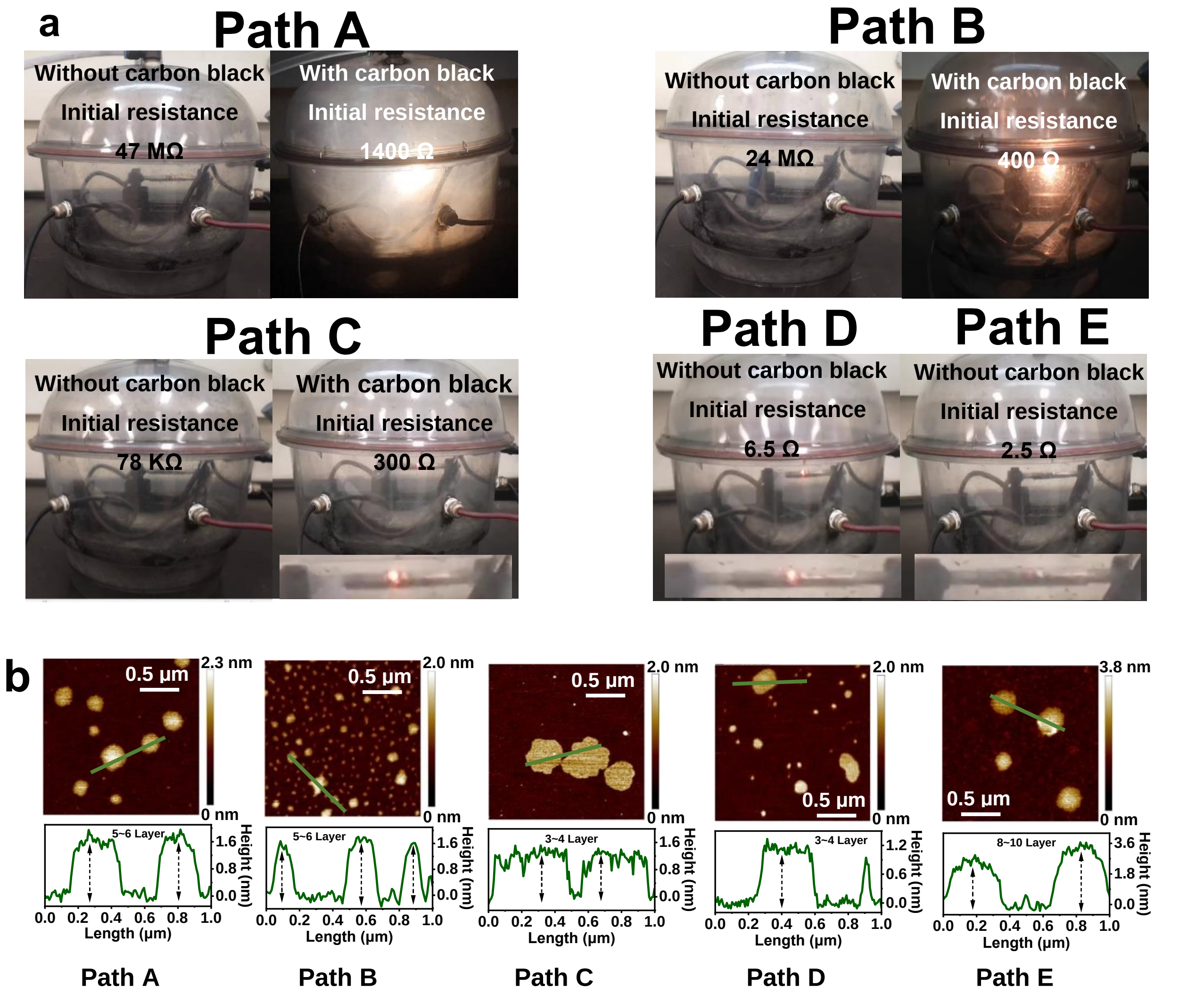

Supplementary Fig. 14 | Biomass-based flash graphene production process. a, Biomass flash graphene production process with/without carbon black from Paths A-E in the alternating current flash Joule heating process. The inner insert images in Path C-E is a magnification of the sample reaction. **b,** AFM of flash graphene from various production paths. Height profile along the dotted green line. Path A: biomass-based flash graphene production; Path B: 300°C biochar-based flash graphene production; Path C: 600°C biochar-based flash graphene production; Path D: 750°C biochar-based flash graphene production; Path E: 900°C biochar-based flash graphene production.

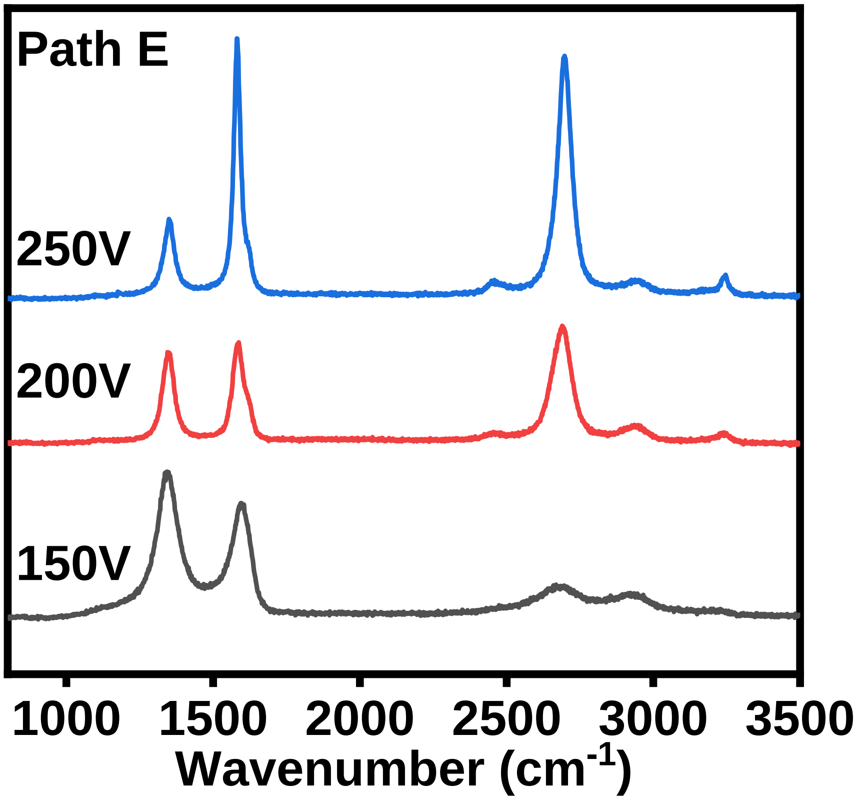

Supplementary Fig. 15 | Raman spectra of flash graphene and graphene-like material from 900°C biochar under various direct current discharge voltages (150, 200, and 250 V). The $I_{2D/G}$ of flash graphene under 250 V exhibit the highest value, which indicated the best few-layer structure among these flash graphene.

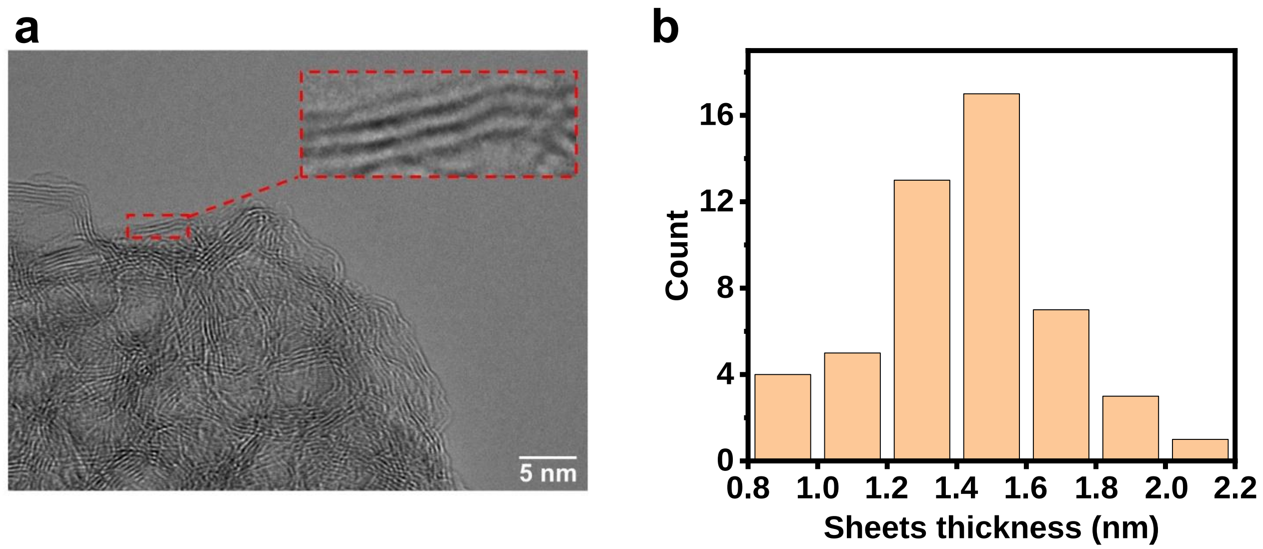

Supplementary Fig. 16 | a, TEM and **b,** sheets thickness (0.8-2.2 nm) of 750°C biochar-based flash graphene. The red dotted line box in the left figure clearly shows that flash graphene with few-layer structure (< 5 layers).

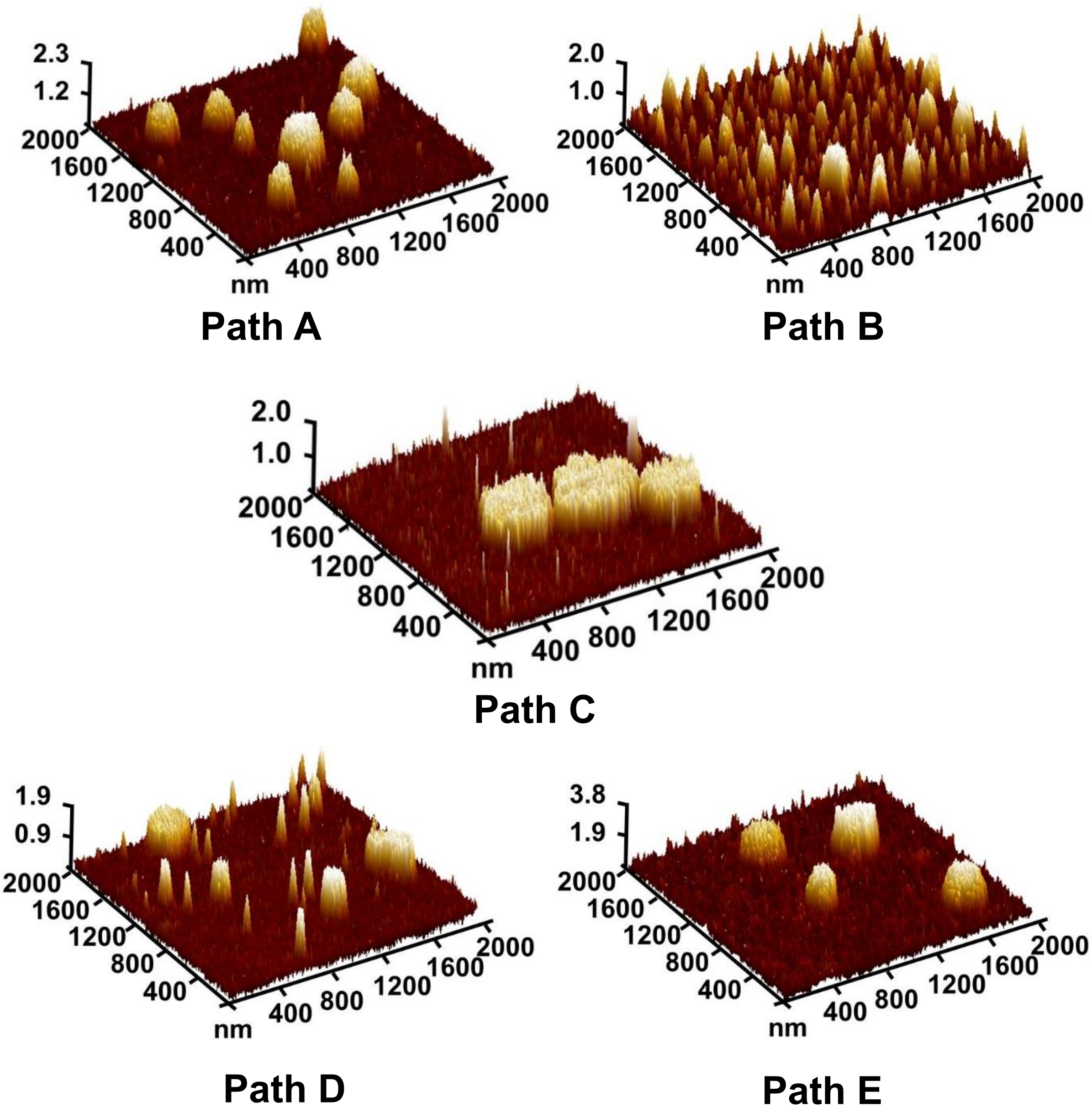

Supplementary Fig. 17 | Three-dimensional AFM diagram of flash graphene from various production path. However, biomass FG fabricated from high-temperature biochar (Path E) exhibits a poor graphene structure like graphite. Path A: biomass-based flash graphene production; Path B: 300°C biochar-based flash graphene production; Path C: 600°C biochar-based flash graphene production; Path D: 750°C biochar-based flash graphene production; Path E: 900°C biochar-based flash graphene production.

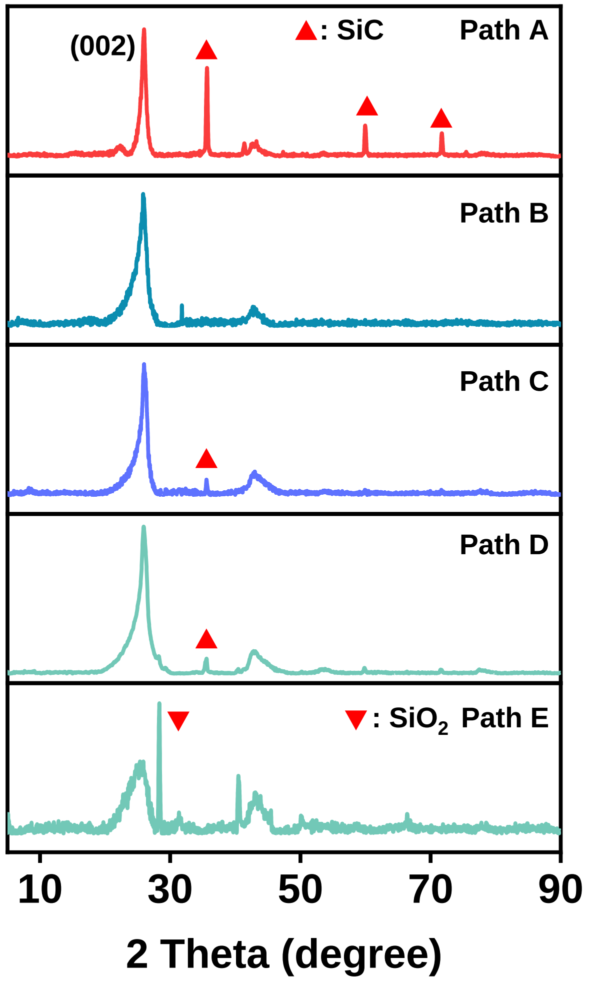

Supplementary Fig. 18 | XRD patterns of flash graphene and graphene-like material from various production paths.

Path A: biomass-based flash graphene production; Path B: 300°C biochar-based flash graphene production; Path C: 600°C biochar-based flash graphene production; Path D: 750°C biochar-based flash graphene production; Path E: 900°C biochar-based flash graphene production.

Supplementary Table 4. The resistance of preliminary FG, resistance ratio of the sample to device, and sample-allocated voltage during DC-FJH of various biomass flash graphene production path.

Pathway	Sample resistance (Ω)	Device resistance (Ω)	Resistance ratio	Allocated voltage (V)
Path A	4.1	0.5	8.2	119
Path B	8.9		17.8	123
Path C	1.7		3.4	84.2
Path D	1.6		3.2	81.2
Path E	1.2		2.4	61.6

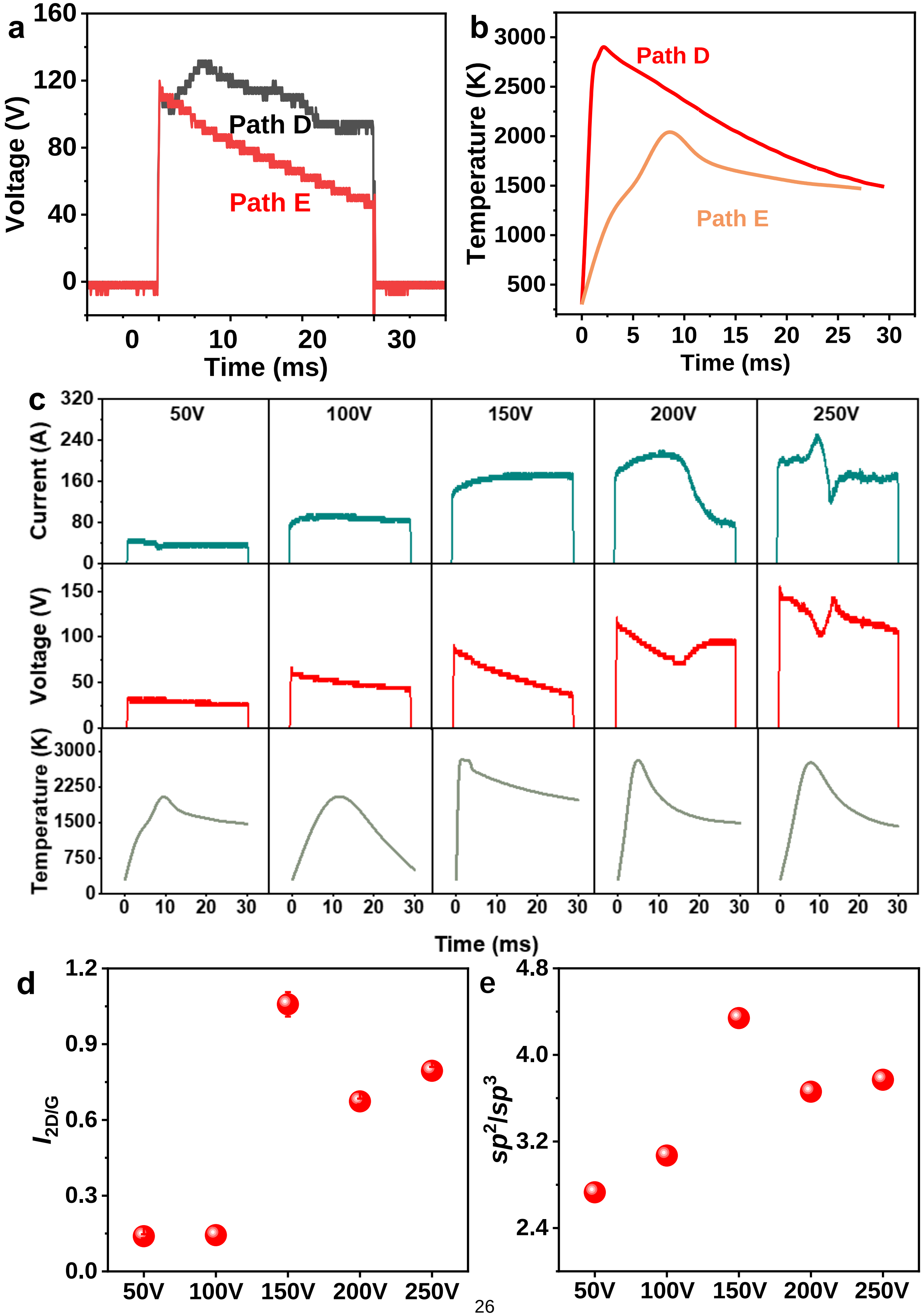
a
b
c
d
e
Voltage (V)
Time (ms)
Path D
Path E
Temperature (K)
50V
100V
150V
200V
250V
Current (A)
Voltage (V)
Temperature (K)
Time (ms)
$I_{2D/G}$
sp^2/sp^3

Supplementary Fig. 19 | Structural regulation of biomass flash graphene. a, Comparison of voltage from 750°C biochar involved flash graphene production (Path D) and 900°C biochar involved flash graphene production (Path E). **b**, Comparison of temperature from 750°C biochar involved flash graphene production (Path D) and 900°C biochar involved flash graphene production (Path E). **c,** Current, voltage, and temperature of 750°C biochar-based flash graphene production under various reaction voltages (50, 100, 150 ,200, and 250 V) were recorded during DC-FJH for 30 ms. **d,** Intensity ratio of the 2D and G bands (in Raman spectra) of 750°C biochar-based flash graphene. Error bars, standard deviations of the results from three samples. **e,** Intensity ratio of sp^2 and sp^3 carbon bond content of the 750°C biochar-based flash graphene. The peaks corresponding to sp^2 carbon (C=C) at 284.57 eV and sp^3 carbon (C-C) at 285.43 eV are fitted by X-ray photoelectron spectroscopy (XPS). And then, sp^2/sp^3 is obtained according to the proportion of peak areas. Error bars, standard deviations of the results from three samples.

Supplementary Table 5. Elemental compositions of preliminary flash graphene derived from various parent materials.

Raw material	C (%)	N (%)	H (%)	S (%)	H/C
Sawdust	82.8	0.12	0.05	0.31	0.007
300°C biochar	79.4	0.32	1.31	0.10	0.198
600°C biochar	95.0	0.14	0.19	0.14	0.024
750°C biochar	92.7	0.32	0.07	0.16	0.009
900°C biochar	86.0	0.40	0.06	0.17	0.008

The hydrogen-carbon ratio is calculated as follows: H/C = (H relative weight/H molecular weight) / (C relative weight /C molecular weight). H relative weight and C relative weight used in this equation are percentage by weight.

Supplementary Table 6. Elemental compositions of flash graphene from various parent materials and various direct current (DC) discharge voltage (50-250 V).

Raw material	DC discharge voltage	C (%)	N (%)	H (%)	S (%)	H/C
Sawdust	150	77.2	0.14	0.96	0.90	0.149
300°C biochar	150	91.7	0.18	0.19	0.27	0.025
600°C biochar	150	94.4	0.13	0.10	0.20	0.013
	50	93.4	0.46	0.05	0.24	0.006
	100	95.2	0.45	0.06	0.20	0.007
750°C biochar	150	96.5	0.11	0.06	0.22	0.008
	200	97.5	0.09	0.04	0.25	0.005
	250	94.3	0.05	0.03	0.22	0.004
900°C biochar	150	91.9	0.21	0.02	0.24	0.003

The hydrogen-carbon ratio is calculated as follows: H/C = (H relative weight/H molecular weight) / (C relative weight /C molecular weight). H relative weight and C relative weight used in this equation are percentage by weight.

Supplementary Table 7. Pyrolysis, AC-FJH, DC-FJH, and total yield of flash graphene production process from various parent materials.

Sawdust	Yield for pyrolysis (%)	Yield for AC-FJH (%)	Yield for DC-FJH (%)	Total yield (%)	Energy/batch (J)
Biomass	-	12.8	82.4	10.5*	1200
300°C	41.3	56.3	77.4	18.0	875.9
600°C	22.4	89.3	88.7	17.7	1500
750°C	21.0	91.8	89.8	17.3	1569
900°C	19.3	87.5	95.2	16.1	1600

*: Loss in Path A is caused by pyrolytic volatiles and depletion. Compared with Path B-E, the yield of a single batch is lower due to the release of pyrolytic volatiles. Therefore, for 1 gram of graphene produced, Path A requires more reaction batches, resulting in a high cumulative loss.

Supplementary Table 8. Life cycle impacts associated with 1 g of flash graphene produced by the five different production systems.

Item	Path A	Path B	Path C	Path D	Path E
Climate change/g CO_2-equiv	10.47	3.751	2.683	2.609	2.598
Fossil depletion/g oil-equiv	2.820	1.910	1.776	1.667	1.651
Terrestrial acidification/g SO_2-equiv	0.028	0.021	0.020	0.020	0.019
Freshwater eutrophication/g P-equiv	0.001	0.001	0.001	0.001	0.001
Metal depletion/g Cu-equiv	0.008	0.003	0.002	0.002	0.002
Particulate matter formation/mg PM2.5-equiv	0.012	0.009	0.009	0.009	0.009
Photochemical oxidant formation/g NO_x-equiv	0.240	0.041	0.015	0.015	0.015
Water depletion/dm^3	0.022	0.021	0.042	0.044	0.046

Path A: biomass-based flash graphene production;

Path B: 300°C biochar-based flash graphene production;

Path C: 600°C biochar-based flash graphene production;

Path D: 750°C biochar-based flash graphene production;

Path E: 900°C biochar-based flash graphene production.

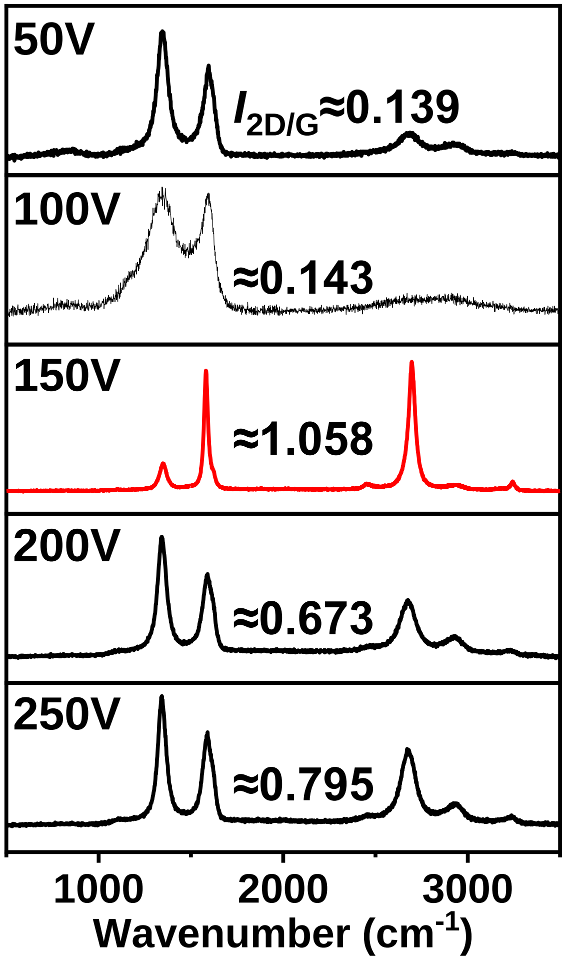

Supplementary Fig. 20 | Raman spectra for flash graphene (150, 200, and 250 V) and graphene-like material (50 and 100 V) under various direct current discharge voltages. The $I_{2D/G}$ of flash graphene under 150 V exhibit the highest value, which indicated the best few-layer structure among these flash graphene.

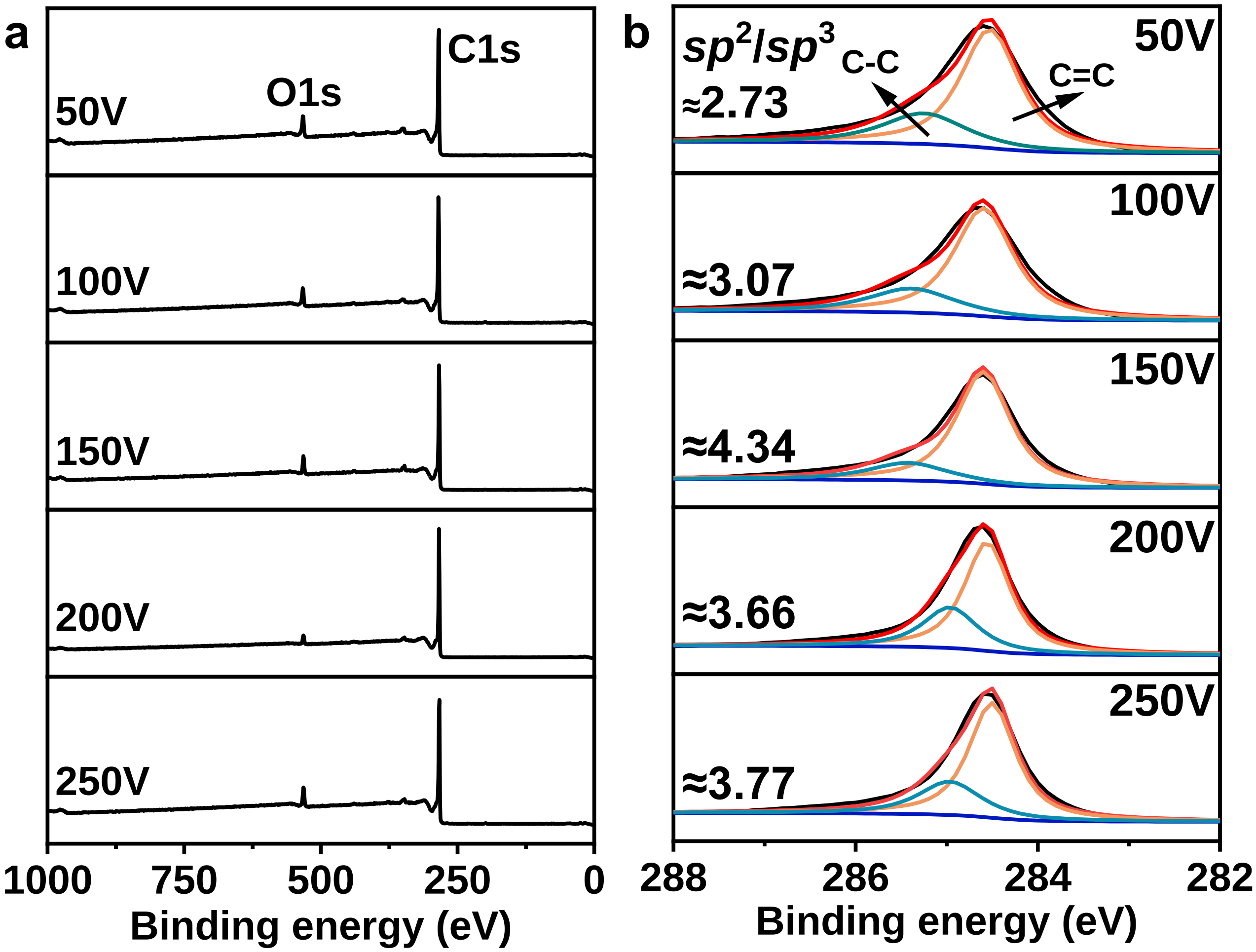

Supplementary Fig. 21 | a, XPS survey scans and **b,** C1 spectra of flash graphene under various direct current discharge voltages (50, 100, 150, 200, and 250 V). The *sp^2/sp^3* of flash graphene under 150 V exhibit the highest value, which indicated the best structure among these flash graphene.

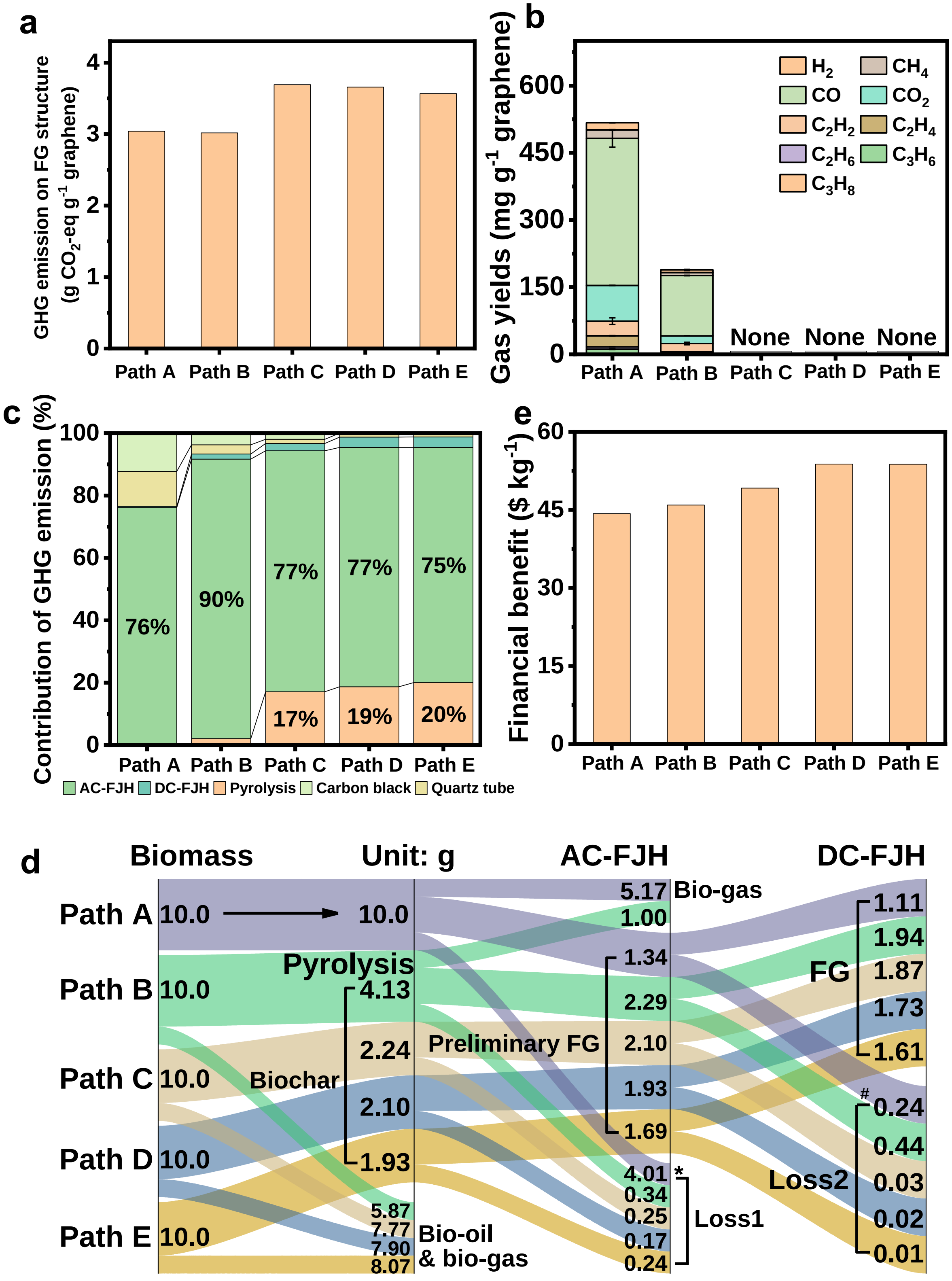

a
GHG emission on FG structure (g CO2-eq g-1 graphene)
Path A
Path B
Path C
Path D
Path E
b
Gas yields (mg g-1 graphene)
H2
CH4
CO
CO2
C2H2
C2H4
C2H6
C3H6
C3H8
None
None
None
c
Contribution of GHG emission (%)
76%
90%
77%
77%
75%
17%
19%
20%
AC-FJH
DC-FJH
Pyrolysis
Carbon black
Quartz tube
e
Financial benefit ($ kg-1)
d
Biomass
Unit: g
AC-FJH
DC-FJH
Path A 10.0
10.0
Path B 10.0
Path C 10.0
Path D 10.0
Path E 10.0
Pyrolysis
4.13
2.24
2.10
1.93
Biochar
5.87
7.77
7.90
8.07
Bio-oil & bio-gas
Preliminary FG
5.17 Bio-gas
1.00
1.34
2.29
2.10
1.93
1.69
4.01
0.34
0.25
0.17
0.24
*
Loss1
FG
1.11
1.94
1.87
1.73
1.61
#
0.24
0.44
0.03
0.02
0.01
Loss2

Supplementary Fig. 22 | Various flash graphene production path and corresponding environmental and economic benefits. a, Required GHG emissions of FG structure optimization during AC-FJH. **b**, Bio-gas yields of each component from Path A and Path B in the AC-FJH process. No volatiles were generated in the AC-FJH process of path C-E produces. Error bars, standard deviations of the results from three samples. **c**, Contribution analysis on the GHG emissions derived from 1 g of flash graphene produced by the five production systems. **d,** Material flow of five different flash graphene production paths from biomass (Path A) or biochar (Path B-E) to flash graphene. "Loss1" refers to the pyrolytic volatiles (bio-oil as main composition) and depletion in AC-FJH. "Loss2" refers to the pyrolytic volatiles (probably bio-oil or bio-gas) and depletion in DC-FJH. *Note: for producing graphene from 10-gram biomass, 100 times AC-FJH reactions are required in Path A, while only 20 times in the Path C-E. Therefore, a high accumulated depletion value is formed in Path A. Overall, a high loss value in path A is formed. #Note: 25 times DC-FJH reactions are required in Path B, while only 16 times in the Path C-E. Therefore, a high accumulated depletion value is formed in Path B. Overall, a high loss value in path B is formed. **e**, Financial benefit of various flash graphene production paths (Path A-E).

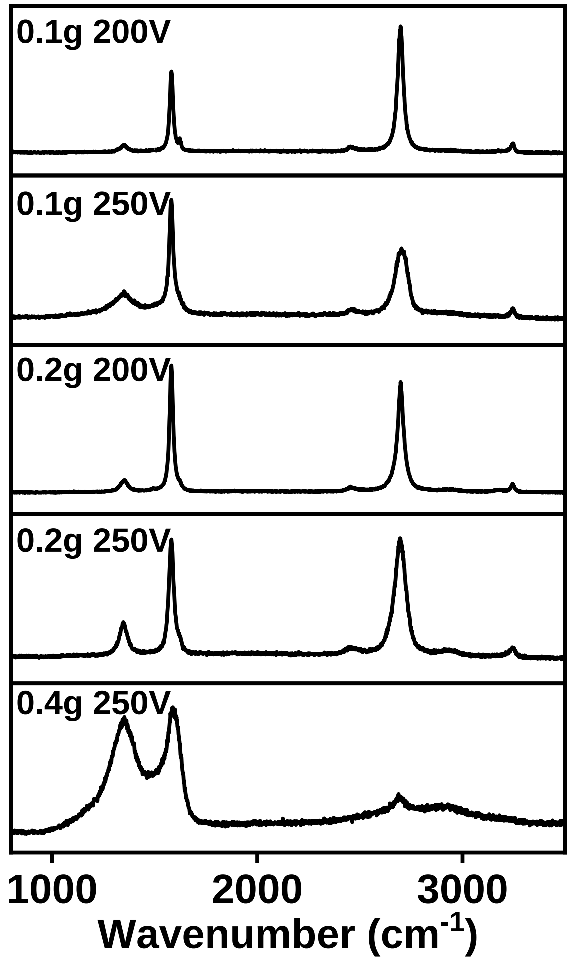

Supplementary Fig. 23 | Raman spectra for 750°C biochar-based flash graphene and graphene-like material from sawdust at pilot-scale with various loading weight (0.1 g, 0.2g, and 0.4g) and various direct current reaction voltage (200 V and 250 V) of samples.

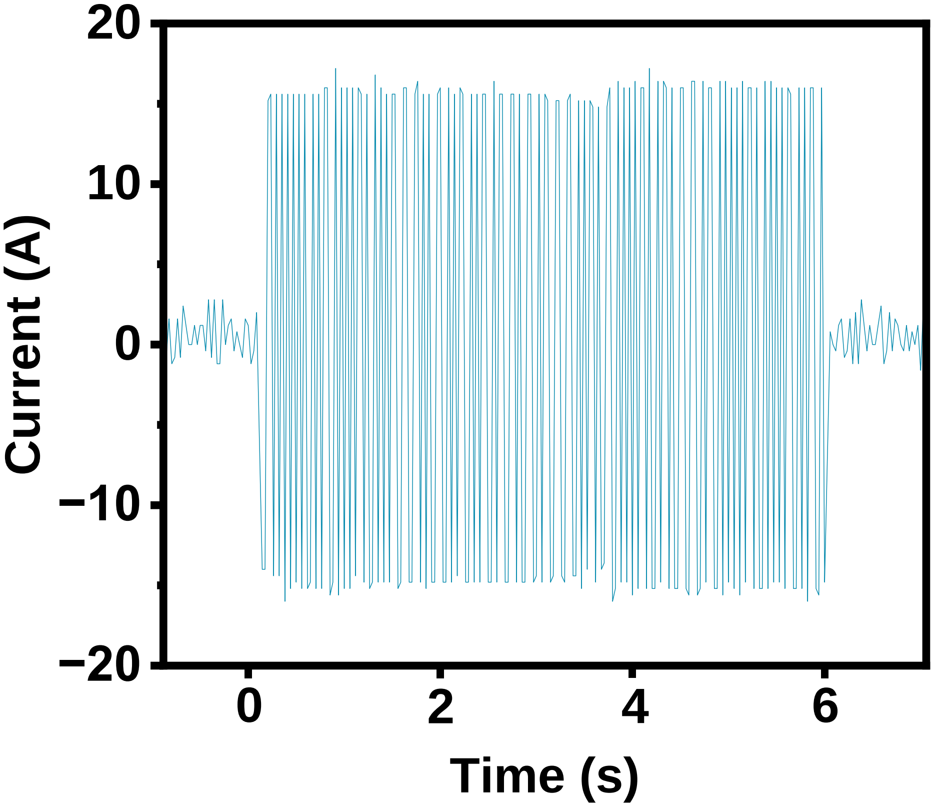

Supplementary Fig. 24 | Current record of one single sample for flash graphene production from sawdust during AC-FJH for 6s at pilot-scale. Due to the ultrafast FJH reaction, there is not enough time to record the current and voltage of the AC-FJH and DC-FJH simultaneously.

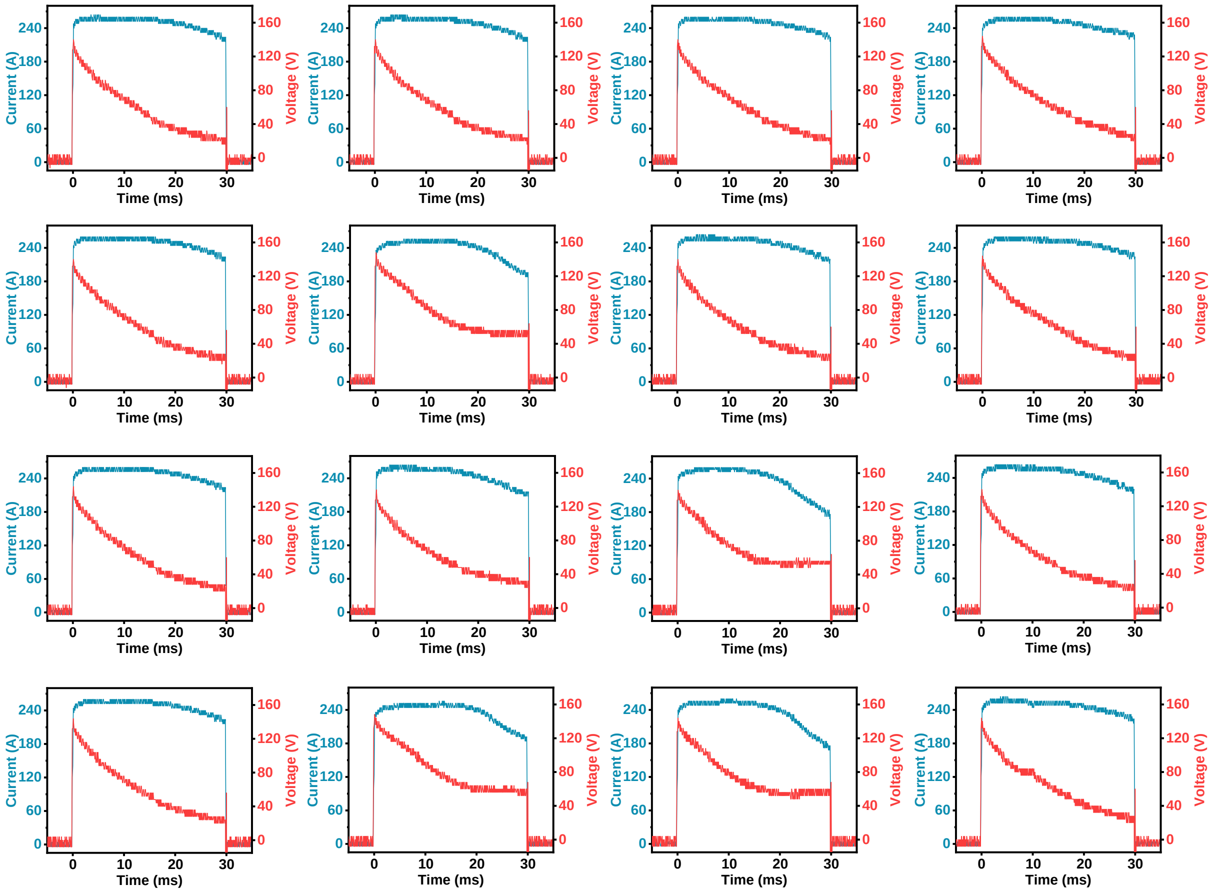

Supplementary Fig. 25 | Continuous records of current and voltage for biochar-based flash graphene production during DC-FJH at pilot-scale (Group 1 of 16 samples).

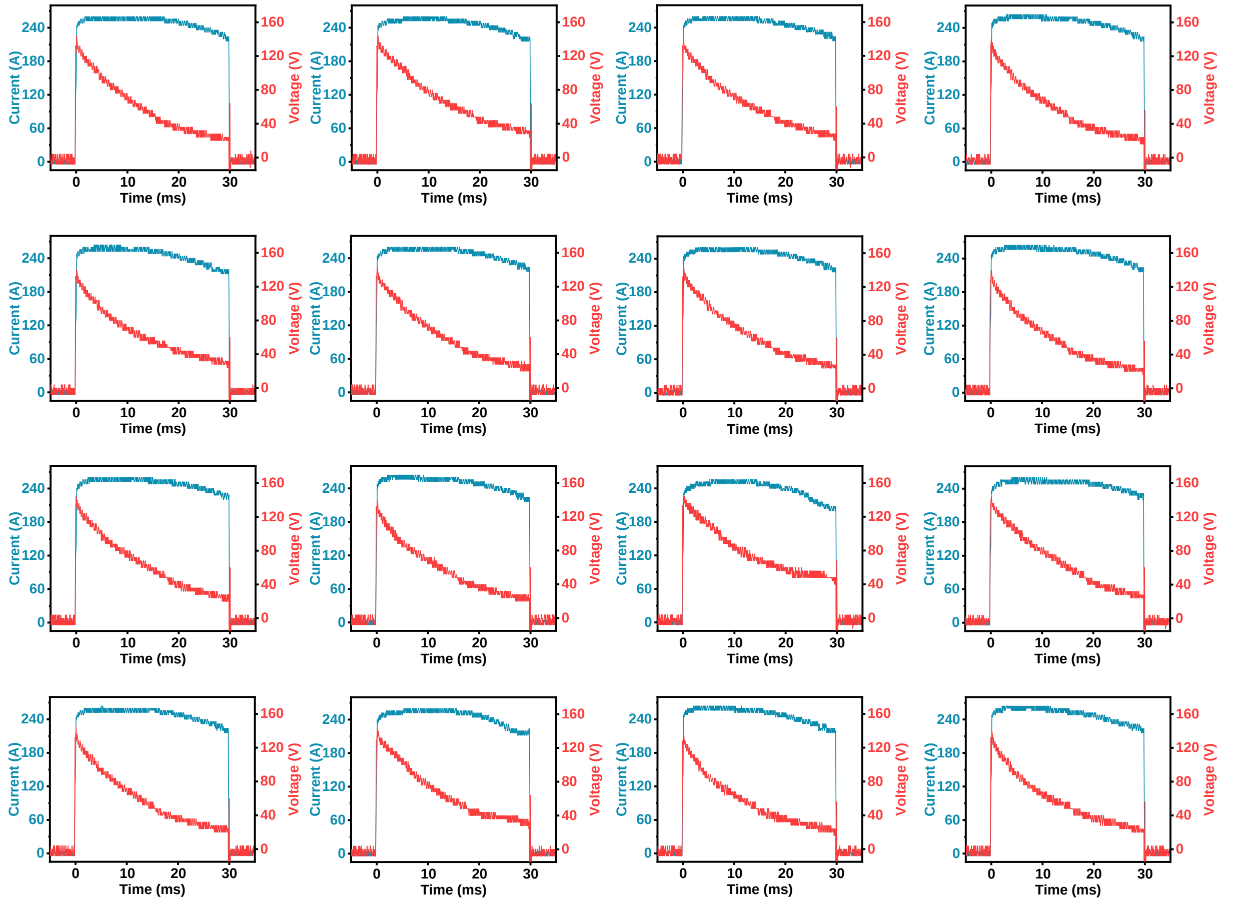

Supplementary Fig. 26 | Continuous records of current and voltage for biochar-based flash graphene production during DC-FJH at pilot-scale (Group 2 of 16 samples).

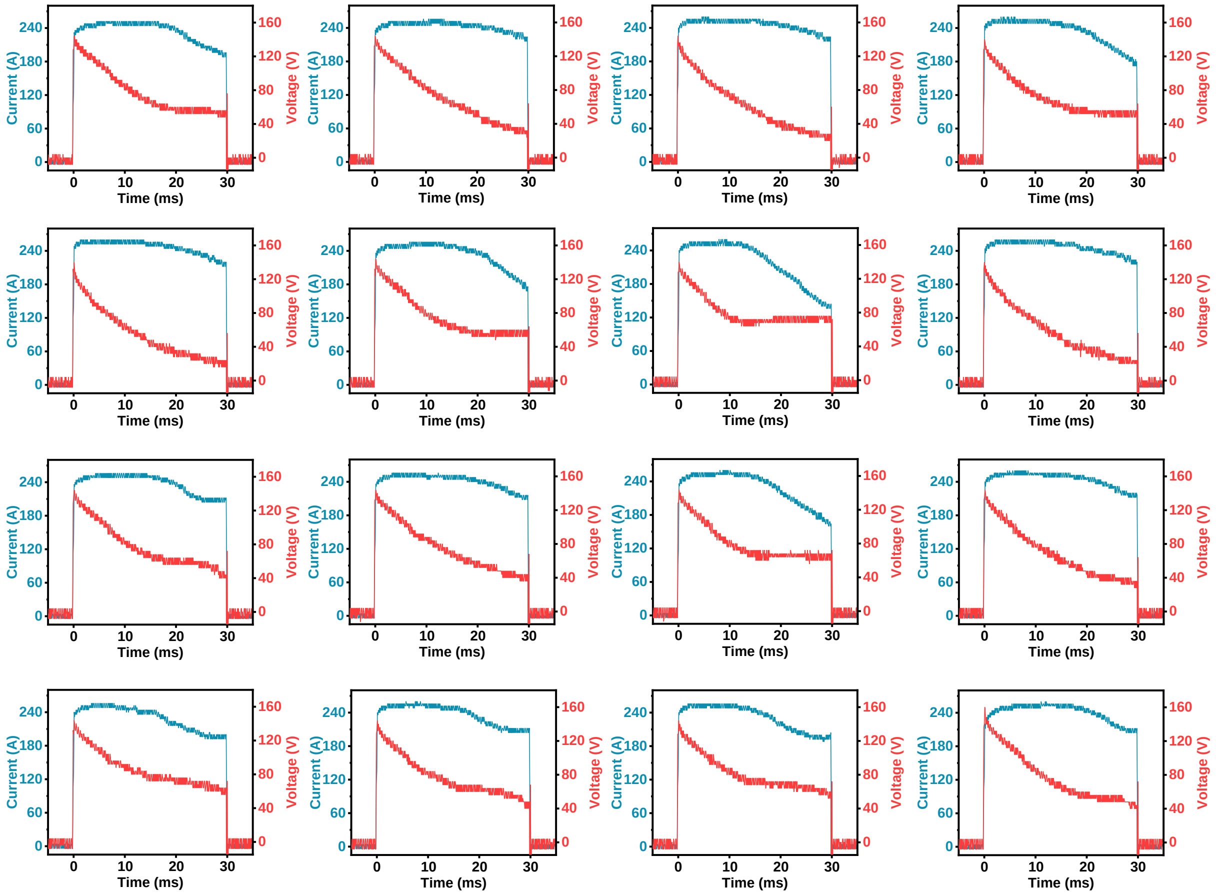

Supplementary Fig. 27 | Continuous records of current and voltage for biochar-based flash graphene production during DC-FJH at pilot-scale (Group 3 of 16 samples).

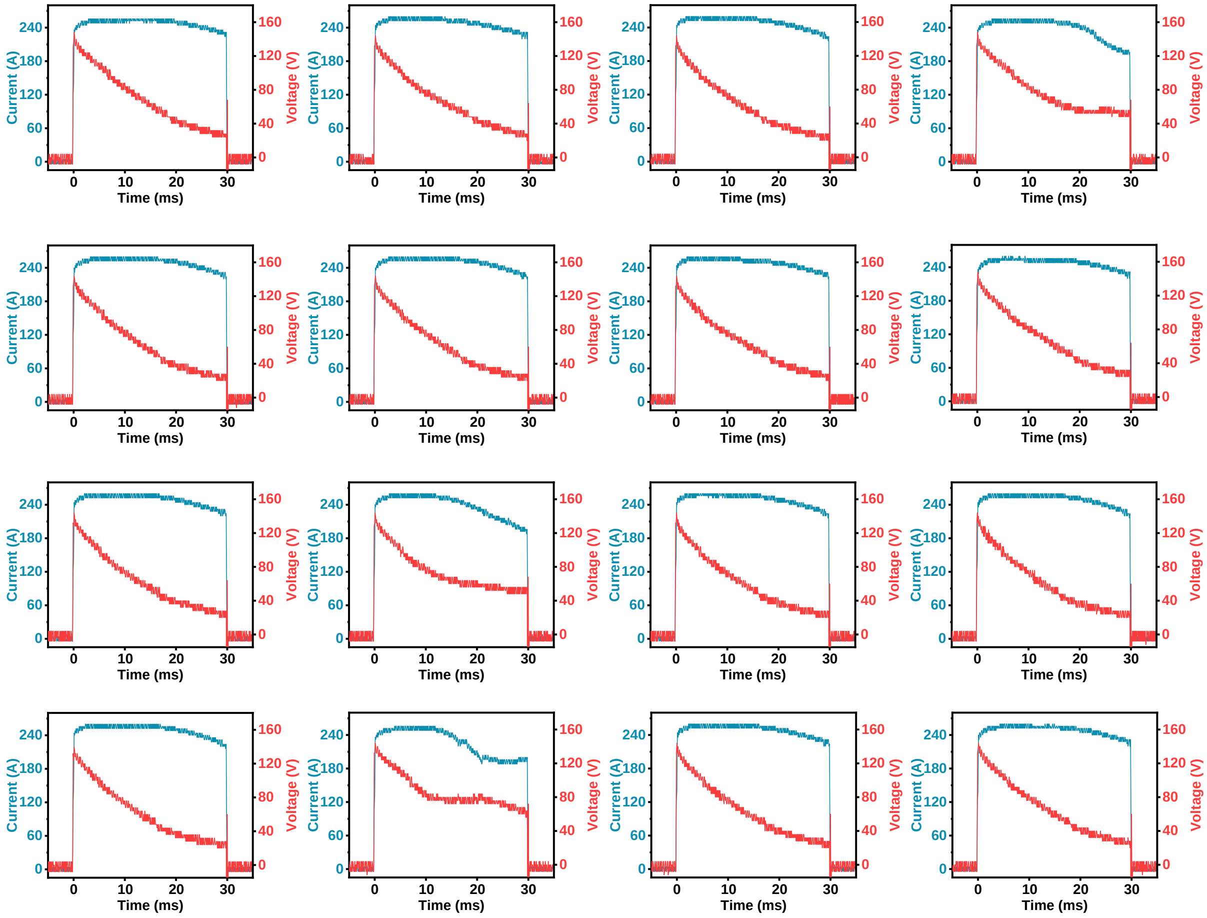

Supplementary Fig. 28 | Continuous records of current and voltage for biochar-based flash graphene production during DC-FJH at pilot-scale (Group 4 of 16 samples).

Supplementary Table 9. Yield of biochar-based flash graphene from sawdust at pilot-scale.

Sample	Yield (%)	Sample	Yield (%)	Sample	Yield (%)	Sample	Yield (%)
1	89.0	17	88.5	33	92.5	49	86.5
2	90.0	18	93.5	34	92.5	50	89.0
3	92.5	19	84.5	35	81.0	51	91.0
4	90.0	20	84.5	36	90.5	52	93.5
5	89.5	21	88.5	37	92.0	53	94.0
6	91.0	22	92.5	38	93.0	54	92.0
7	90.0	23	93.0	39	92.5	55	91.0
8	88.0	24	90.5	40	86.5	56	89.0
9	89.0	25	91.0	41	89.0	57	84.5
10	91.5	26	89.5	42	88.5	58	87.5
11	89.0	27	90.0	43	91.5	59	89.0
12	92.0	28	90.5	44	86.5	60	88.5
13	90.5	29	91.5	45	91.0	61	89.0
14	92.0	30	86.0	46	96.0	62	93.0
15	92.5	31	91.5	47	91.0	63	85.5
16	84.5	32	90.5	48	90.5	64	87.0

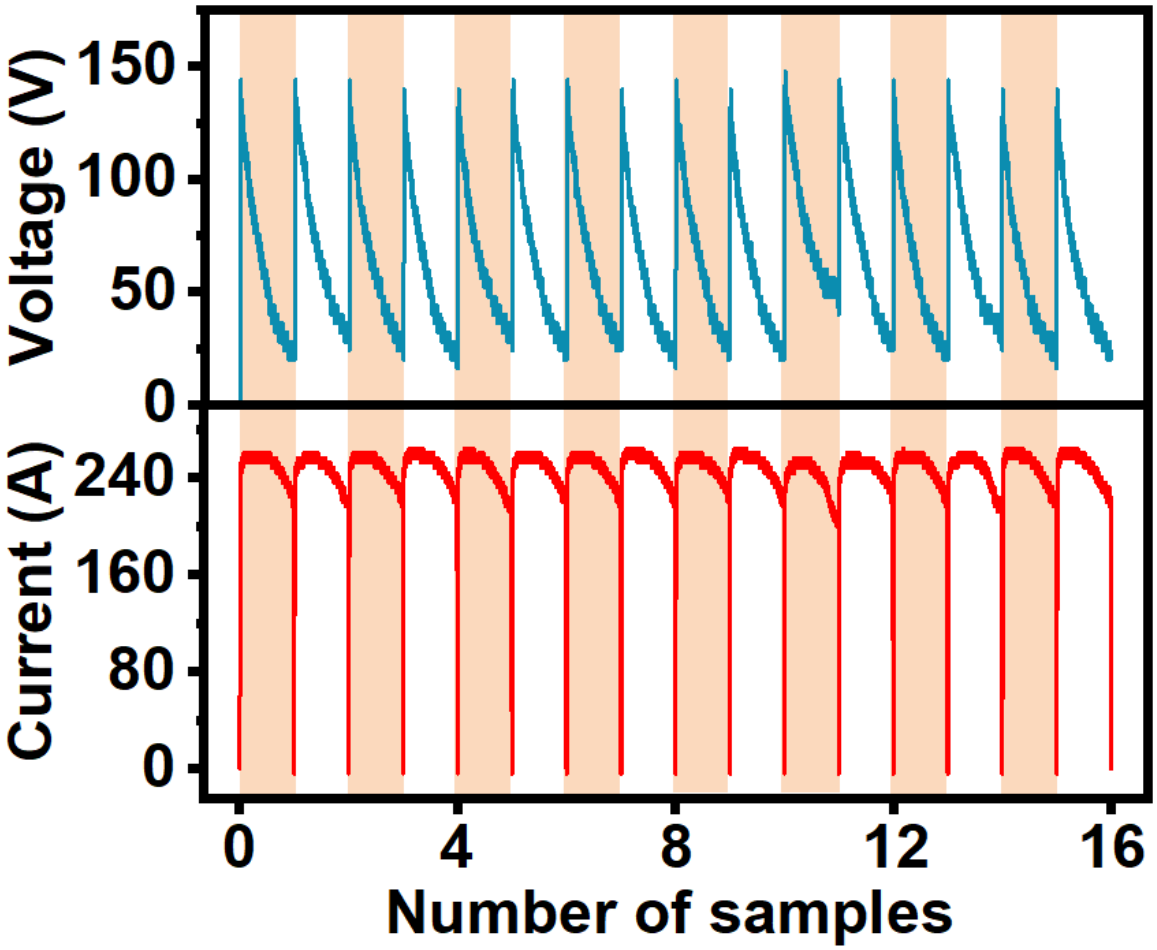

Supplementary Fig. 29 | Continuous records of current and voltage for biochar-based flash graphene from sawdust production during DC-FJH at pilot-scale for one group (16 samples). Pink-highlighted area is the corresponding current and voltage to FJH reaction of one single sample.

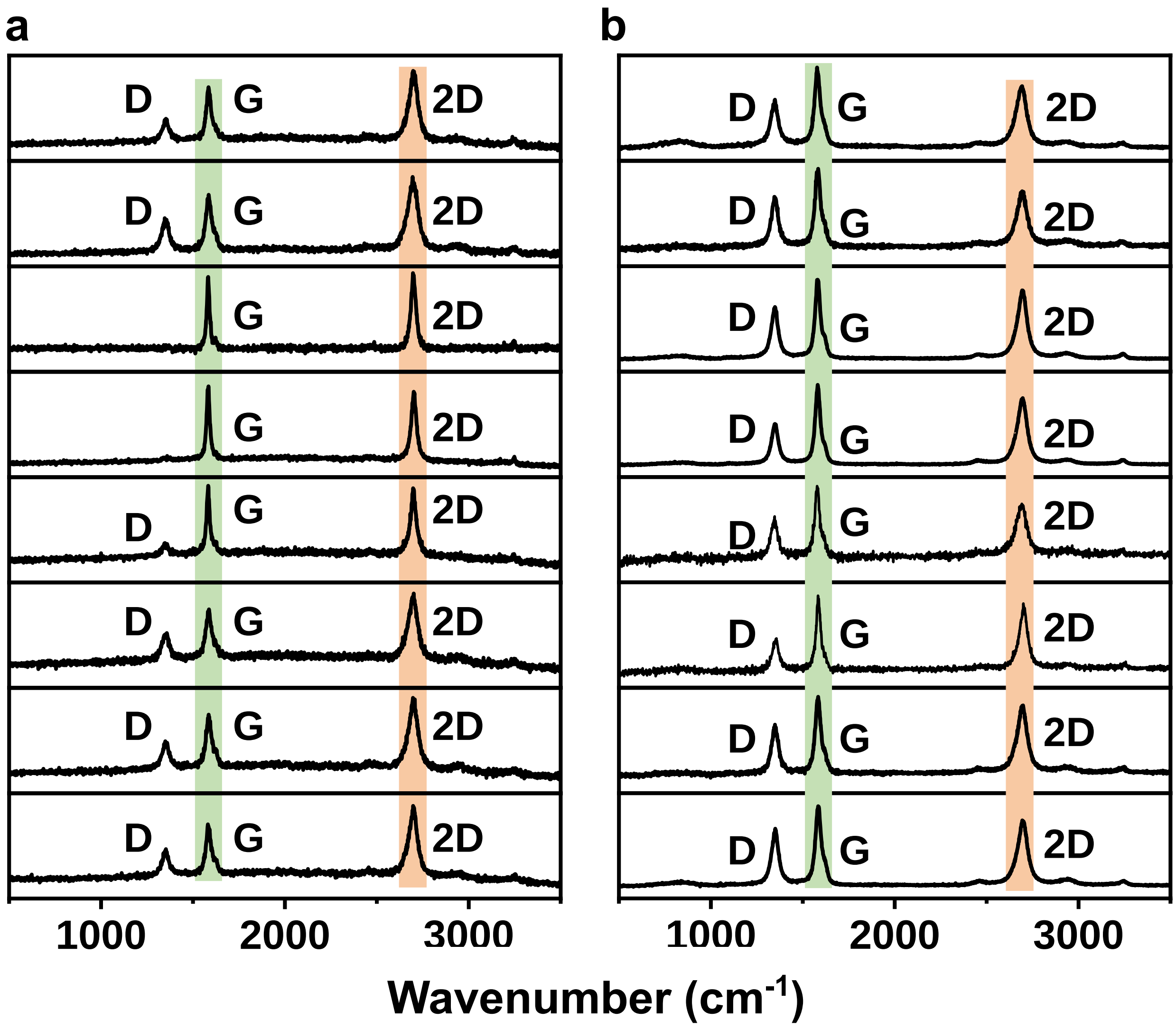

Supplementary Fig. 30 | Raman spectra of biochar-based few-layer flash graphene from sawdust at pilot-scale for samples **a,** 1 to 8 and **b,** 9 to 16, which had similar ratio of 2D band and G band.

Supplementary Table 10. Intensity value of the D/G and 2D/G (in Raman spectra) of biochar-based flash graphene from sawdust at pilot-scale.

Sample	$I_{D/G}$	$I_{2D/G}$
1	0.531	1.366
2	0.603	1.272
3	0.000	0.951
4	0.072	0.929
5	0.200	0.992
6	0.565	1.323
7	0.514	1.300
8	0.526	1.367
9	0.590	0.762
10	0.633	0.710
11	0.647	0.863
12	0.501	0.829
13	0.580	0.739
14	0.419	0.899
15	0.636	0.889
16	0.719	0.818

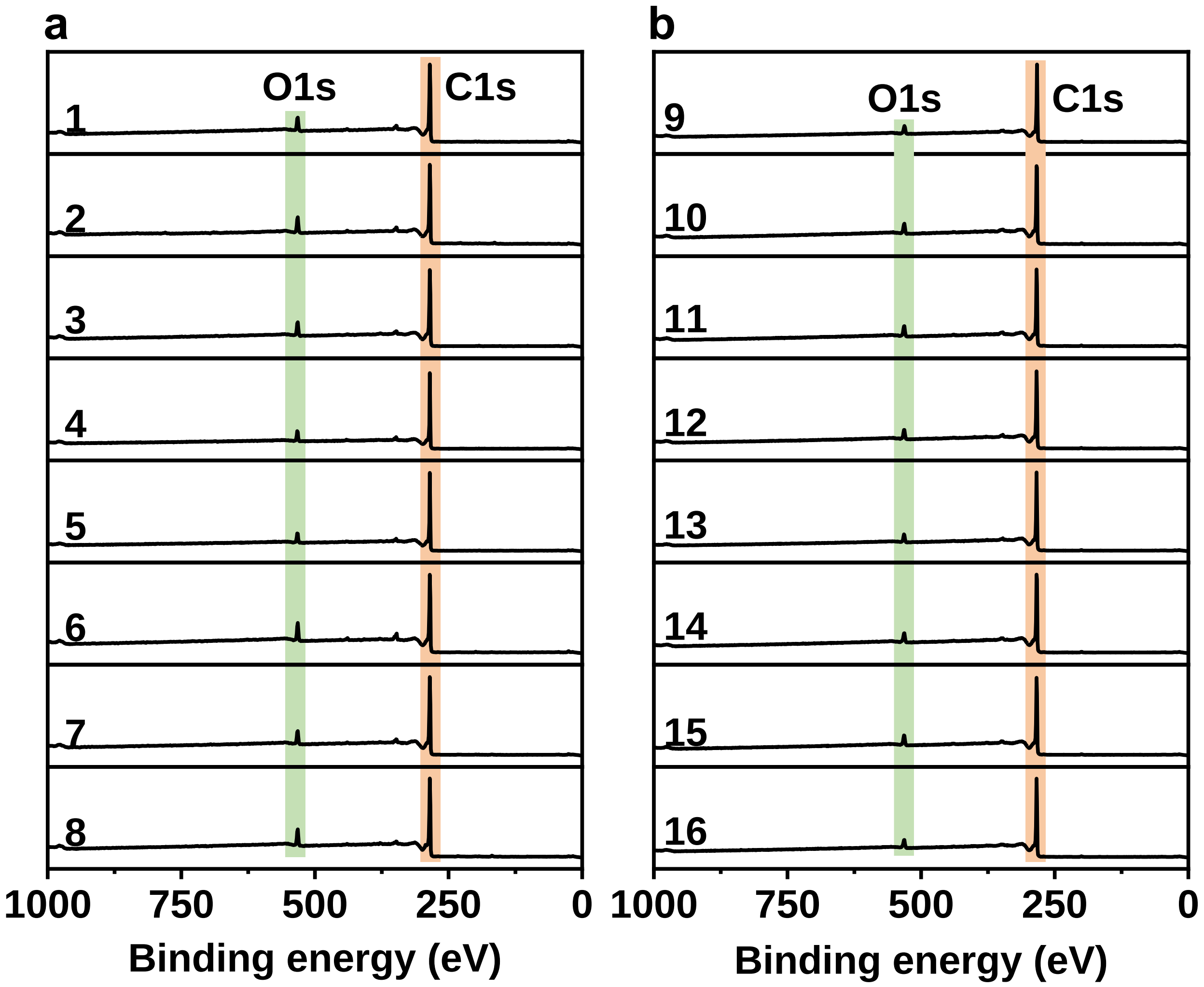

Supplementary Fig. 31 | XPS survey scans of biochar-based flash graphene (**a,** samples 1 to 8 and **b,** 9 to 16) at pilot-scale, which shows ultralow impurity.

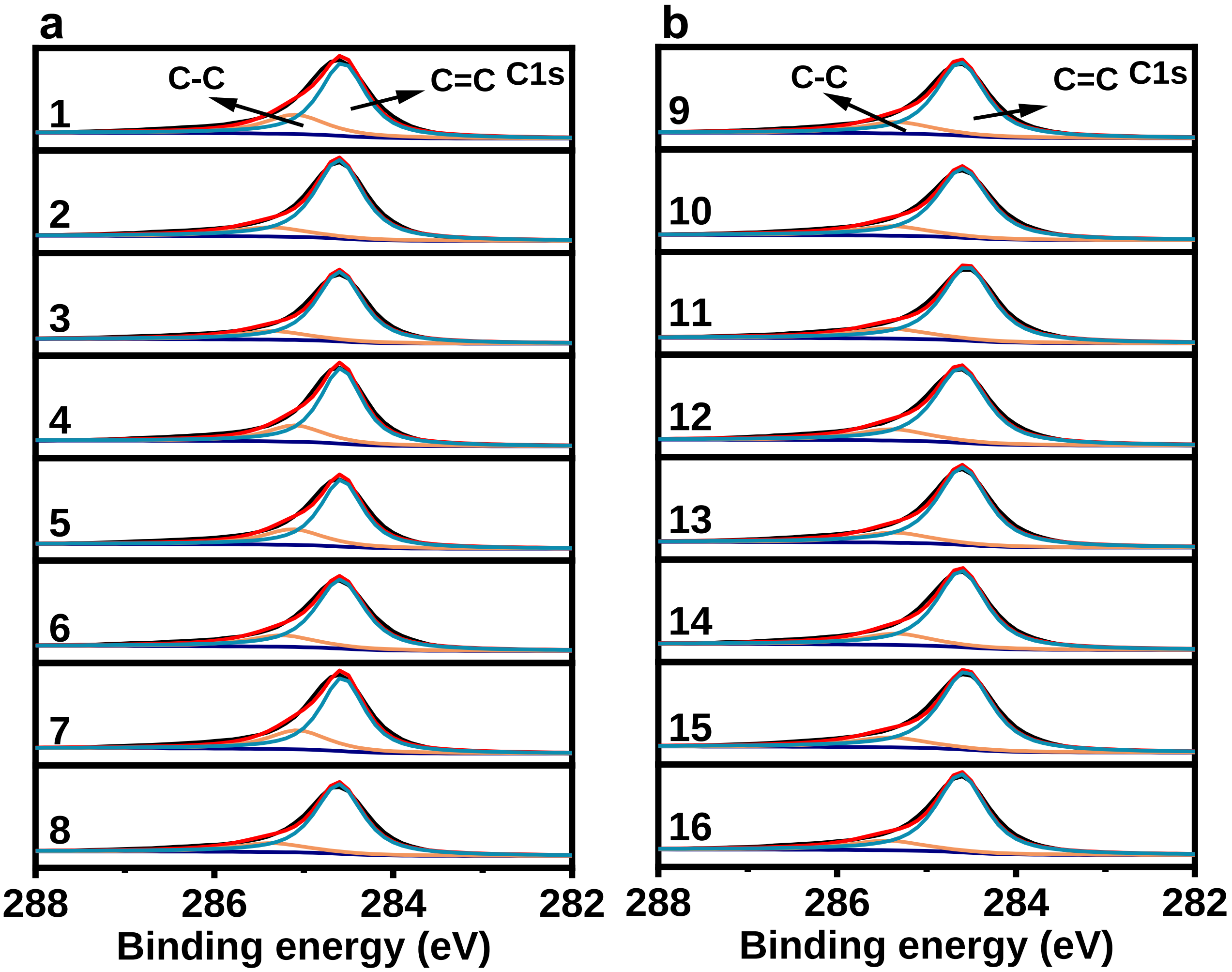

Supplementary Fig. 32 | C 1S XPS spectra of biochar-based flash graphene (**a,** samples 1 to 8 and **b,** 9 to 16) from sawdust at pilot-scale, which shows stable value of sp^2/sp^3. The binding energies of high resolutions spectra were calibrated using the C 1s peak at 284.8 eV.

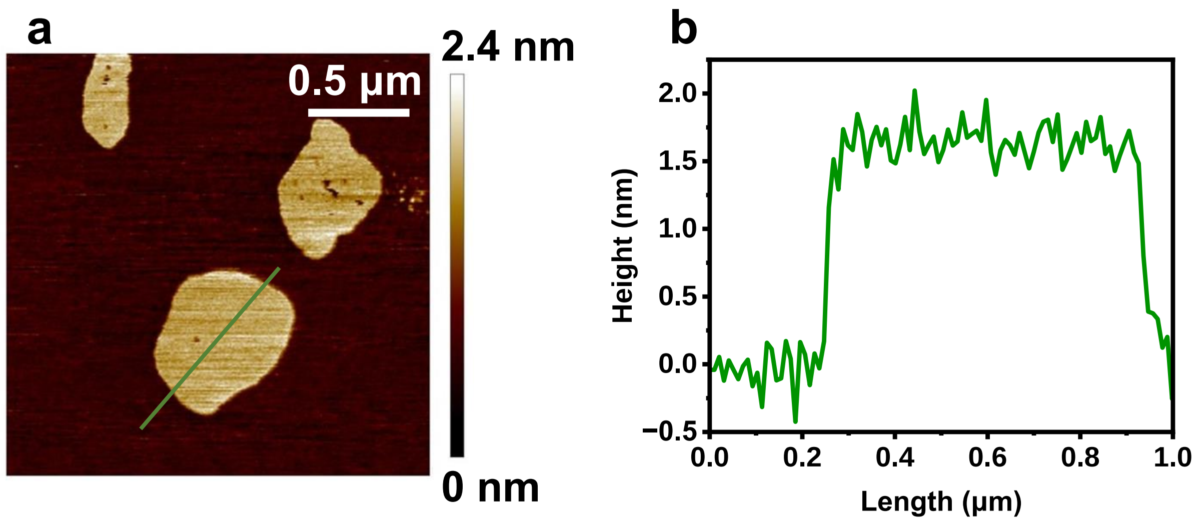

Supplementary Fig. 33 | **a**, Two-dimension AFM and **b**, height diagram of biochar-based flash graphene from sawdust at pilot-scale. The AFM diagram demonstrated that biomass flash graphene consisted of 4-5 layers graphene structure.

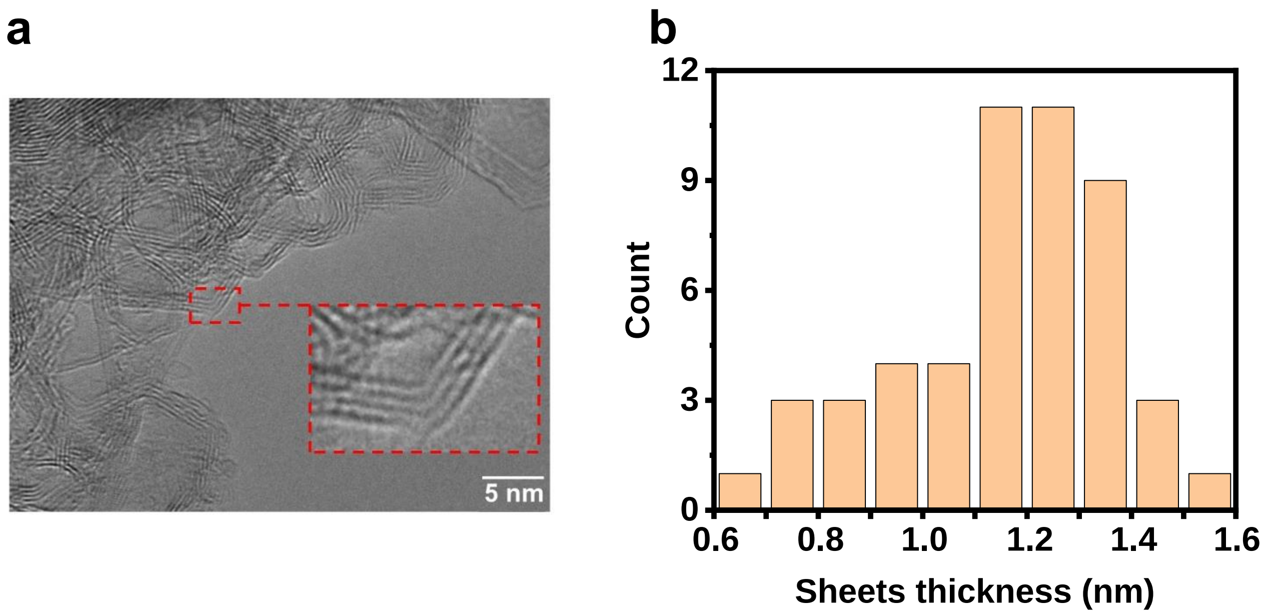

Supplementary Fig. 34 | a, TEM and **b,** sheets thickness of biochar-based flash graphene from sawdust at pilot-scale. The red box in the left figure clearly shows that flash graphene with few-layer structure (< 5 layers).

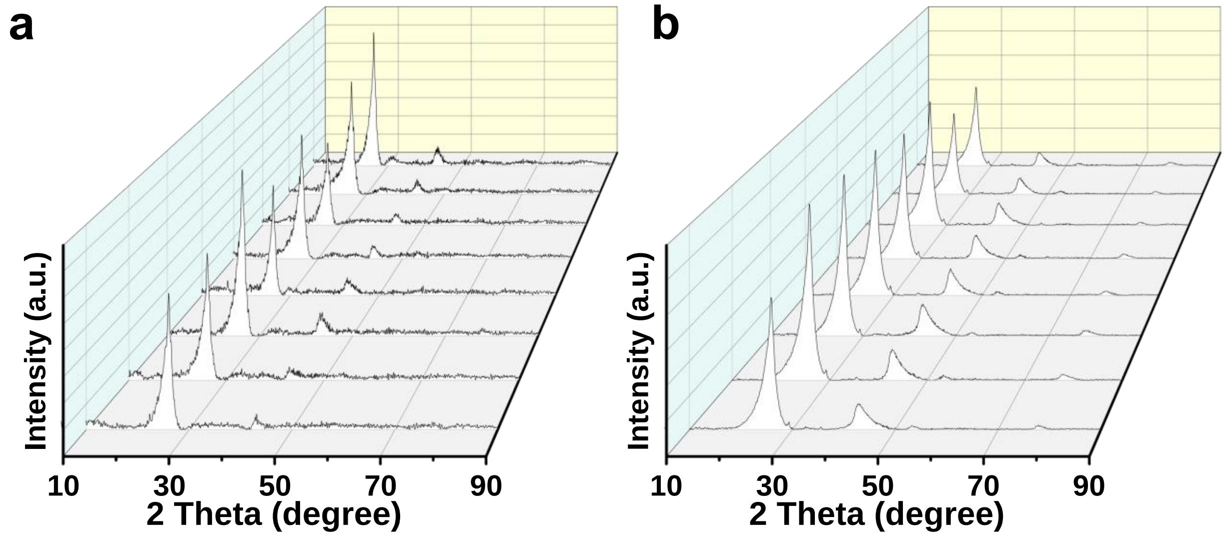

Supplementary Fig. 35 | XRD patterns of sawdust flash graphene from 16 batches (**a,** batches 1 to 8 and **b,** 9 to 16) at pilot-scale. It was indicated that produced biomass flash graphene at pilot-scale contain low impurity.

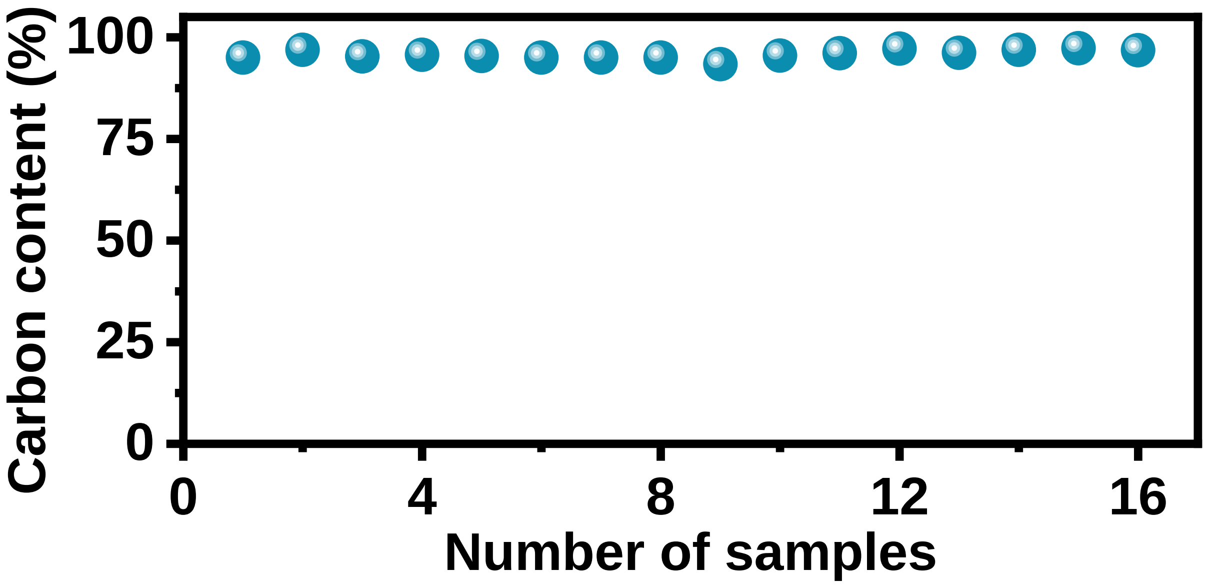

Supplementary Fig. 36 | Carbon content of biochar-based flash graphene from sawdust at pilot-scale. The elemental analysis (C/H/N/S) of FG was performed using an elemental analyzer (Vario EL III, Germany).

Supplementary Table 11. Elemental compositions (C/H/N/S) of biochar-based flash graphene from sawdust at pilot-scale.

Sample	C (%)	N (%)	H (%)	S (%)	H/C
1	95.0	0.14	0.06	0.15	0.008
2	96.9	0.14	0.07	0.18	0.008
3	95.3	0.14	0.05	0.09	0.006
4	95.7	0.15	0.03	0.05	0.004
5	95.4	0.15	0.05	0.10	0.006
6	95.0	0.14	0.03	0.07	0.004
7	95.0	0.16	0.03	0.08	0.004
8	95.0	0.15	0.03	0.04	0.003
9	93.4	0.08	0.05	0.55	0.006
10	95.5	0.10	0.04	0.34	0.005
11	96.1	0.10	0.04	0.34	0.005
12	97.2	0.09	0.02	0.28	0.003
13	96.2	0.08	0.02	0.21	0.002
14	96.9	0.08	0.01	0.18	0.001
15	97.3	0.08	0.01	0.11	0.001
16	96.8	0.09	0.03	0.06	0.004

The hydrogen-carbon ratio is calculated as follows: H/C = (H relative weight/H molecular weight) / (C relative weight /C molecular weight). H relative weight and C relative weight used in this equation are percentage by weight.

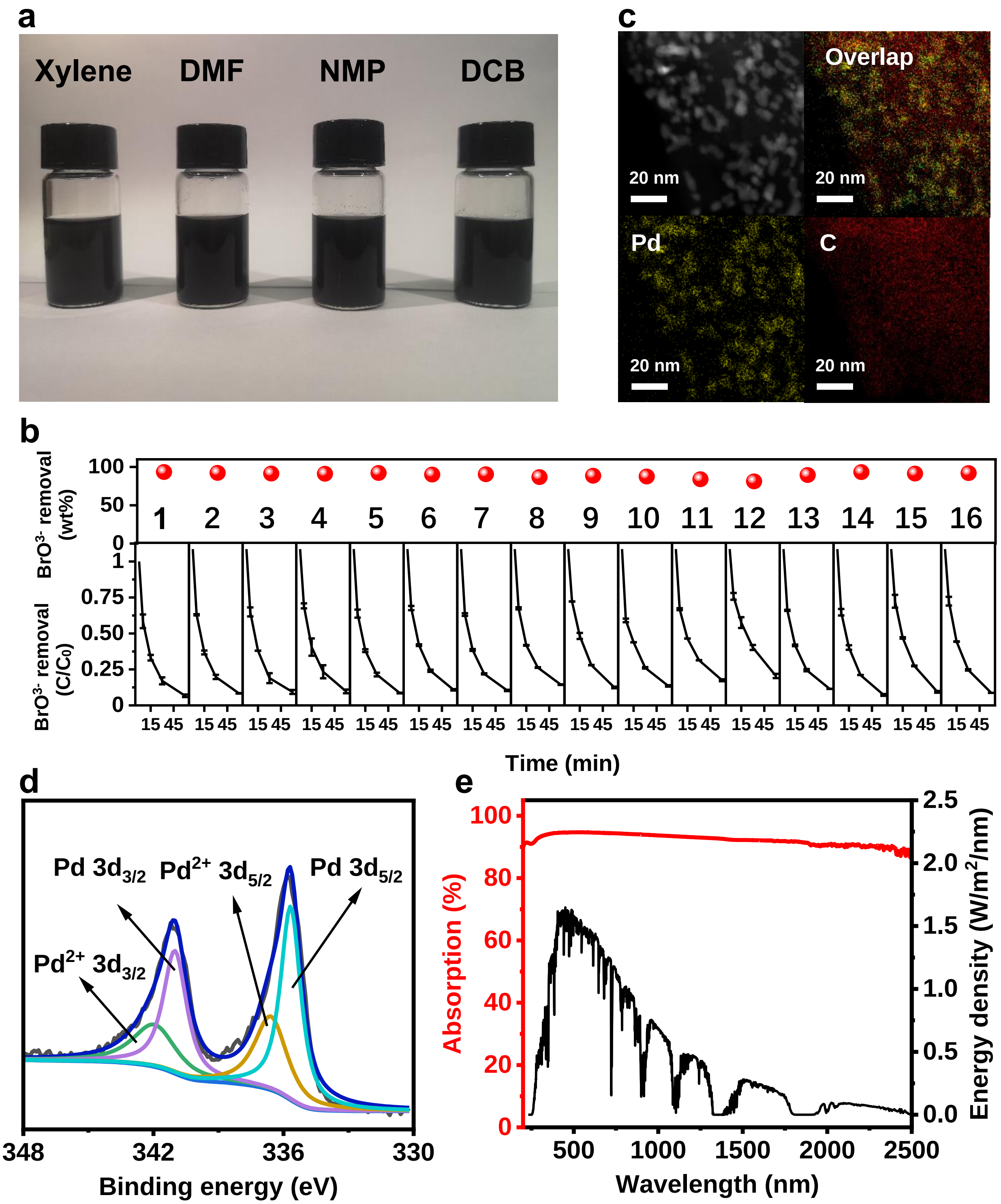

a
Xylene
DMF
NMP
DCB
c
Overlap
20 nm
Pd
C
b
BrO^{3-} removal (wt%)
100
50
0
1 2 3 4 5 6 7 8 9 10 11 12 13 14 15 16
BrO^{3-} removal (C/C_0)
1
0.75
0.50
0.25
15 45
Time (min)
d
Pd $3d_{3/2}$
Pd^{2+} $3d_{5/2}$
Pd $3d_{5/2}$
Pd^{2+} $3d_{3/2}$
348
342
336
330
Binding energy (eV)
e
Absorption (%)
100
80
60
40
20
0
Energy density ($W/m^2/nm$)
2.5
2.0
1.5
1.0
0.5
0.0
500
1000
1500
2000
2500
Wavelength (nm)

Supplementary Fig. 37 | Application performance of biochar-based flash graphene at pilot-scale. a, Flash graphene from pilot-scale dispersion in various organic solvents at 5 g L^{-1} (xylene, N, N-Dimethylformamide (DMF), N-Methyl pyrrolidone (NMP), 3,3'-dichlorobenzidine dihydrochloride (DCB)). **b**, Kinetic analysis of BrO^{3-} removal over 16 batches of biochar-based flash graphene at pilot-scale. The experiments were repeated twice. **c,** High-angle annular dark-field (HAADF) images and corresponding energy-dispersive X-ray (EDX) elemental mapping of Pd, C, and their overlapped images of Pd loaded biochar-based flash graphene. **d,** High-resolution of XPS of Pd 2p of Pd loaded biochar-based flash graphene. **e,** Absorption spectra and solar energy density of biochar-derived flash graphene over the wavelength range of 250-2500 nm.

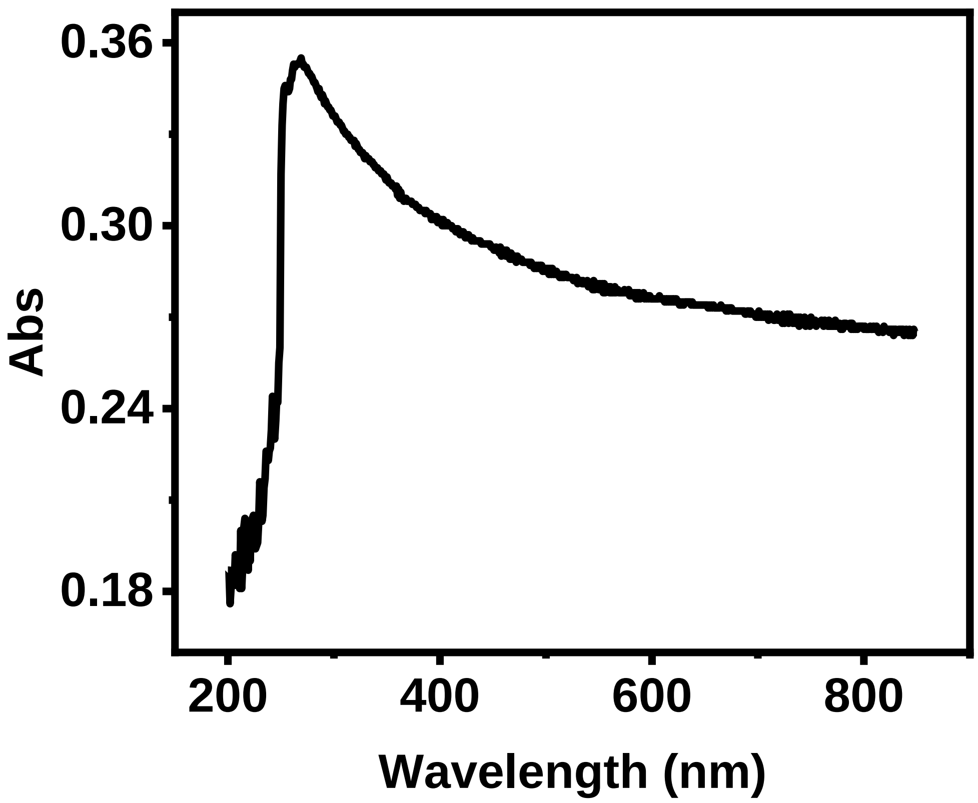

Supplementary Fig. 38 | Wavelength scanning of water–Pluronic (F-127) solution from 200 to 850 nm with 1 nm step size. The maximum absorption wavelength was located in 269 nm. Therefore, the dispersibility of biomass flash graphene in F-127 was measured at 269 nm.

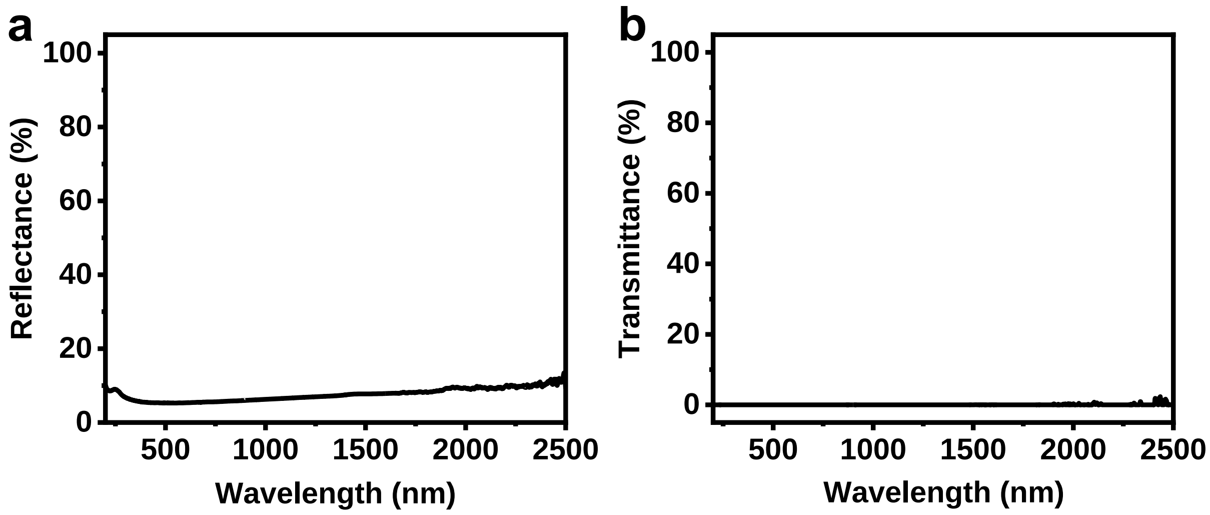

Supplementary Fig. 39 | a, Reflectance and **b,** transmittance spectra of 750 °C biochar-derived flash graphene from sawdust at pilot-scale over the wavelength range of 250 ~ 2500 nm. A small amount of light is reflected and almost no light is transmitted.

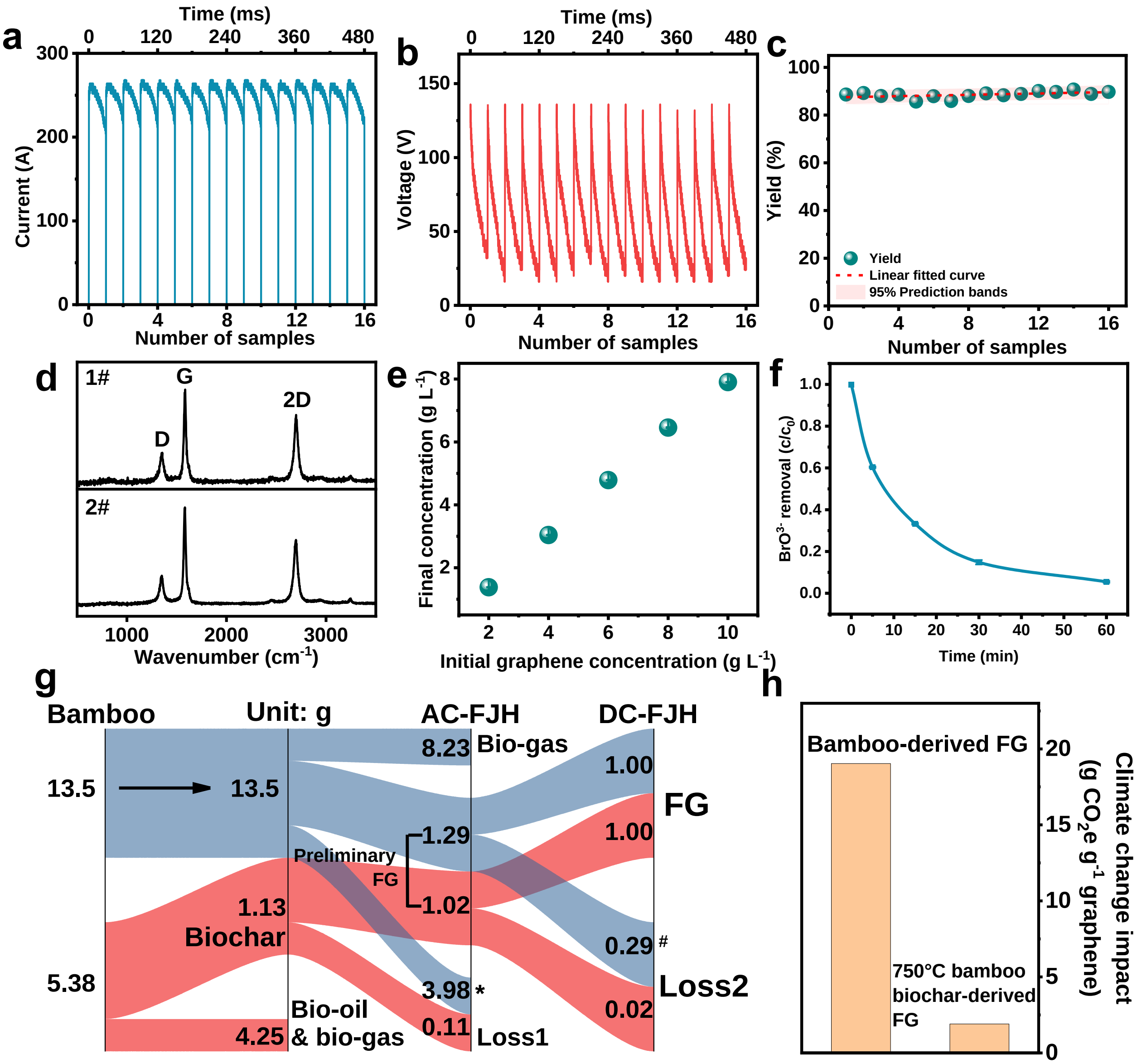

Supplementary Fig. 40 | Production, structural analysis, and application of biochar-based flash graphene from bamboo at pilot-scale. Continuous 16 samples current (**a**) and voltage (**b**) record of 750 °C biochar-based flash graphene production during DC-FJH. **c,** Measured yield of 16 samples of biochar-based flash graphene. Red dotted line is linear fitted curve fitted to the sample yield. Pink-highlighted area is the fitted 95% prediction bands according to the linear fitted curve. **d,** Raman spectra for two mixed biochar-based flash graphene. **e,** Flash graphene dispersion with various concentration ranges from 2-10 mg L^{-1}. The experiments were repeated twice. **f,** Kinetic analysis of

BrO^{3-} removal of biochar-based flash graphene. Error bars, standard deviations of the results from two samples. **g,** Material flow of biomass-based flash graphene and 750 °C biochar-based flash graphene production. “Loss1” refers to the pyrolytic volatiles (bio-oil as main composition) and depletion in AC-FJH. “Loss2” refers to the pyrolytic volatiles (probably bio-oil or bio-gas) and depletion in DC-FJH. *Note: for 1 gram graphene production, 139 times AC-FJH reactions are required in biomass-derived production path, while only 12 times in the biochar-derived production path. Therefore, a high accumulated depletion value is formed biomass-derived production path. Overall, a high loss value (3.98) is formed. #Note: 13 times DC-FJH reactions are required in biomass-derived production path, while 9 times in the biochar-derived production path. Therefore, a high accumulated depletion value is formed in biomass-derived production path. Overall, a high loss value in biomass-derived production path is formed. **h,** Comparison of life cycle GHG emissions between biomass-based flash graphene system and 750 °C biochar-based flash graphene system.

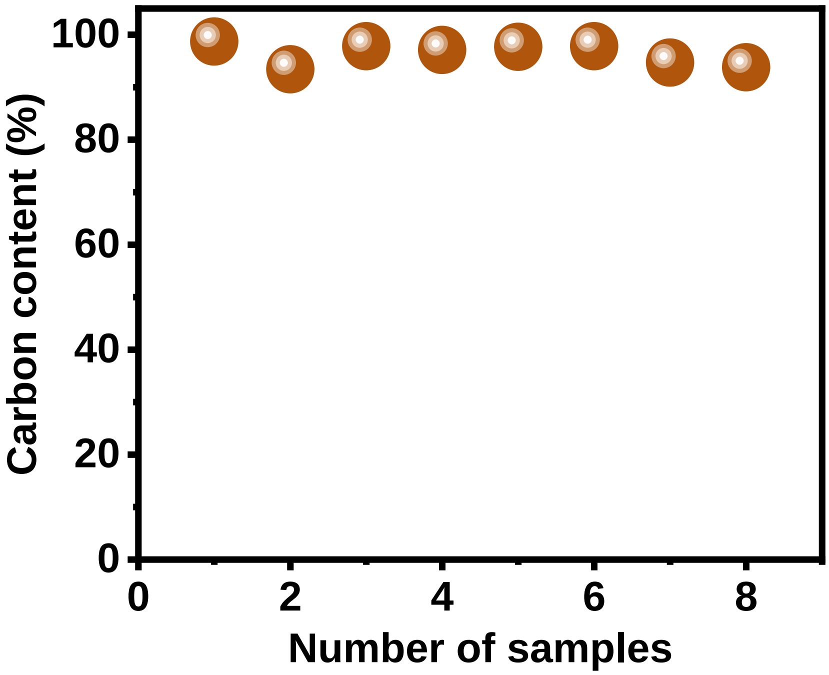

Supplementary Fig. 41 | Carbon content of 750 °C biochar-based flash graphene from bamboo at pilot-scale, which containing ultralow impurity.

Supplementary Table 12. Elemental compositions (C/H/N/S) of biochar-based flash graphene from bamboo at pilot-scale.

Sample	C (%)	N (%)	H (%)	S (%)	H/C
1	98.7	0.24	0.02	0.06	0.002
2	93.4	0.19	0.02	0.08	0.002
3	97.8	0.09	0.00	0.18	0.000
4	97.1	0.20	0.01	0.16	0.001
5	97.7	0.16	0.01	0.19	0.001
6	97.8	0.16	0.01	0.17	0.001
7	94.7	0.24	0.00	0.12	0.000
8	93.8	0.14	0.00	0.16	0.000

The hydrogen-carbon ratio is calculated as follows: H/C = (H relative weight/H molecular weight)/ (C relative weight /C molecular weight). H relative weight and C relative weight used in this equation are percentage by weight.

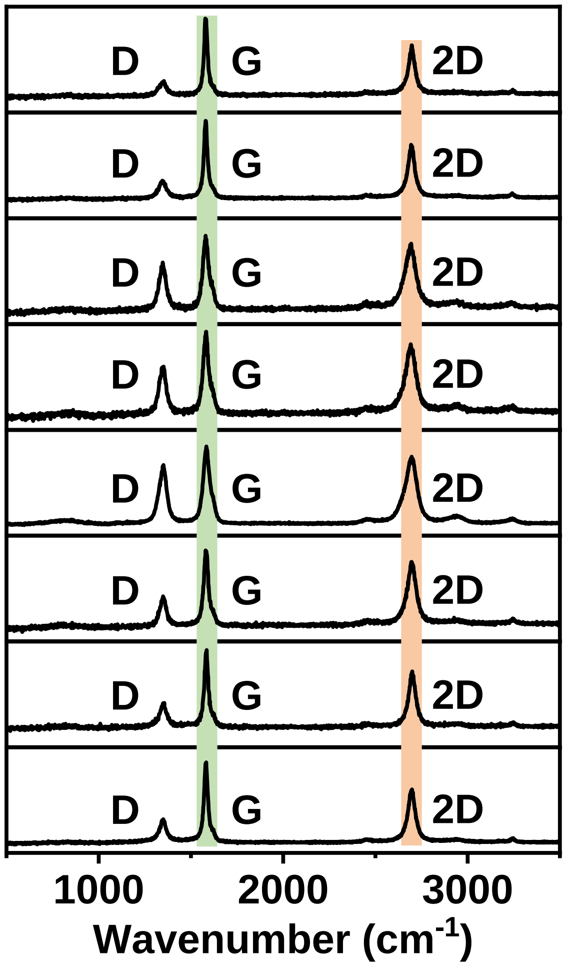

Supplementary Fig. 42 | Raman spectra of biochar-based flash graphene from bamboo at pilot-scale for eight samples, which had similar ratio of 2D band and G band.

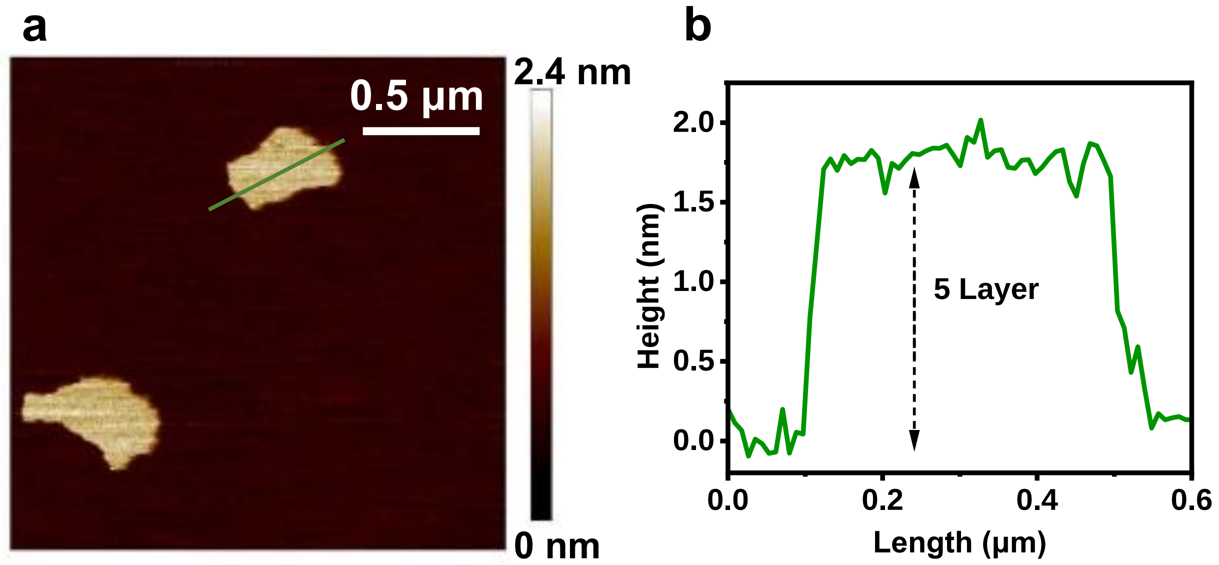

Supplementary Fig. 43 | a, AFM of biochar-based flash graphene from bamboo at pilot-scale. **b,** Height profile along the dotted green line.

Supplementary Table 13. Elemental compositions of preliminary flash graphene derived from bamboo, bamboo derived 750°C biochar, and rice straw derived 750°C biochar. Parent materials were operated on alternating current (200 V) for 6 s and then direct voltage discharge on 150 V for 30 ms.

Raw material	C (%)	N (%)	H (%)	S (%)	H/C
Bamboo	80.0	0.18	0.46	0.11	0.068
Bamboo derived 750°C biochar	86.4	0.34	0.06	0.04	0.009
Rice straw derived 750°C biochar	54.9	0.84	0.08	0.38	0.017

The hydrogen-carbon ratio is calculated as follows: H/C = (H relative weight/H molecular weight) / (C relative weight /C molecular weight). H relative weight and C relative weight used in this equation are percentage by weight.

Supplementary Table 14. Elemental compositions of flash graphene derived from bamboo, bamboo derived 750°C biochar, and rice straw derived 750°C biochar. Parent materials were operated on alternating current (200 V) for 6 s and then direct voltage discharge on 150 V for 30 ms.

Raw material	C (%)	N (%)	H (%)	S (%)	H/C
Bamboo	86.8	0.14	0.18	0.39	0.024
Bamboo derived 750°C biochar	93.6	0.14	0.06	0.19	0.008
Rice straw derived 750°C biochar	56.4	0.61	0.07	0.65	0.014

The hydrogen-carbon ratio is calculated as follows: H/C = (H relative weight/H molecular weight) / (C relative weight /C molecular weight). H relative weight and C relative weight used in this equation are percentage by weight.

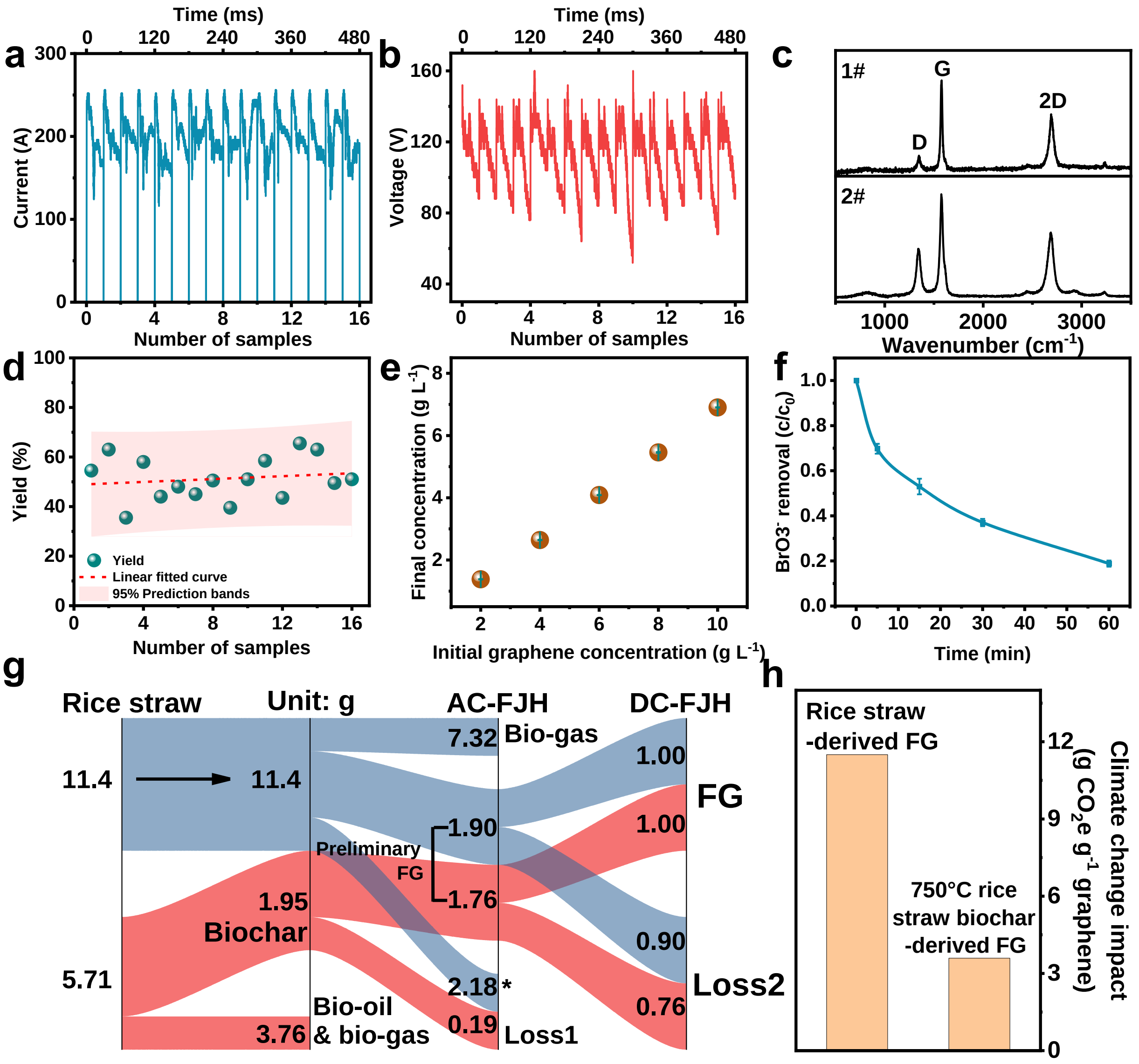

Supplementary Fig. 44 | Production, structural analysis, and application of biochar-based flash graphene from rice straw at pilot-scale. Continuous 16 samples current (**a**) and (**b**) record of 750 °C biochar-based flash graphene production during DC-FJH. **c,** Raman spectra for two mixed biochar-based flash graphene. **d,** Measured yield of 16 samples of biochar-based flash graphene. Red dotted line is linear fitted curve fitted to the sample yield. Pink-highlighted area is the fitted 95% prediction bands according to the linear fitted curve. **e,** flash graphene dispersion with various concentration ranges from 2-10 mg L^{-1}. The experiments were repeated twice. **f,** Kinetic analysis of BrO^{3-} removal of

biochar-based flash graphene. Error bars, standard deviations of the results from two samples. **g,** Material flow of biomass-based flash graphene and 750 °C biochar-based flash graphene production. “Loss1” refers to the pyrolytic volatiles (bio-oil as main composition) and depletion in AC-FJH. “Loss2” refers to the pyrolytic volatiles (probably bio-oil or bio-gas) and depletion in DC-FJH. *Note: for 1 gram graphene production, 114 times AC-FJH reactions are required in biomass-derived production path, while only 20 times in the biochar-derived production path. Therefore, a high accumulated depletion value is formed biomass-derived production path. Overall, a high loss value (2.18) is formed. **h,** Comparison of life cycle GHG emissions between biomass-based flash graphene system and 750 °C biochar-based flash graphene system.

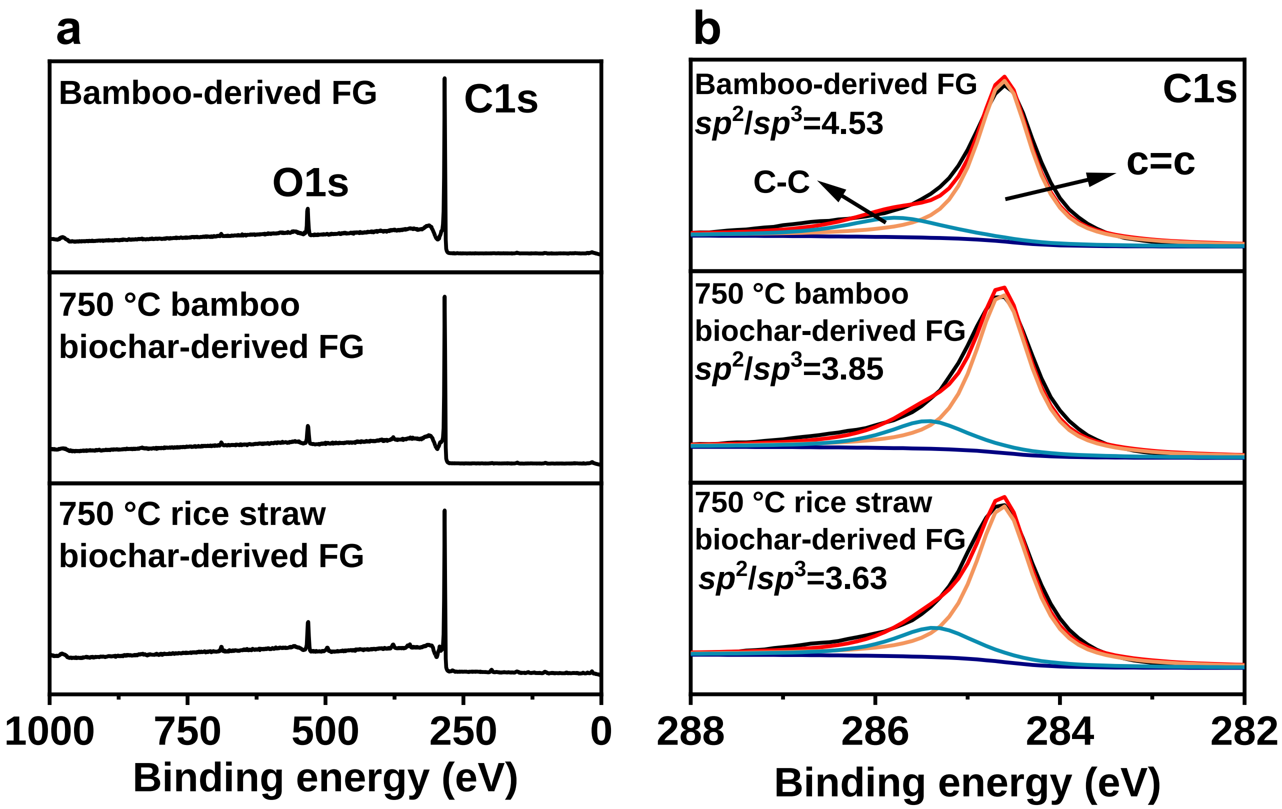

Supplementary Fig. 45 | a, XPS survey scans and **b,** C1 spectra XPS spectra of flash graphene from bamboo, bamboo-based 750 °C biochar, and rice straw-based 750 °C biochar, respectively.

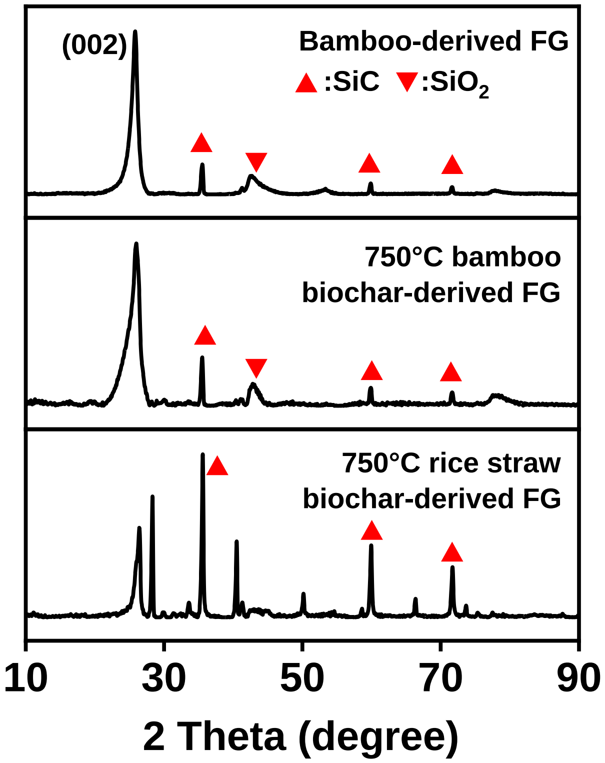

Supplementary Fig. 46 | XRD patterns of flash graphene from bamboo, bamboo-based 750 °C biochar, rice straw-based, and 750 °C biochar, respectively. Rice straw biochar-based flash graphene present many impurities.

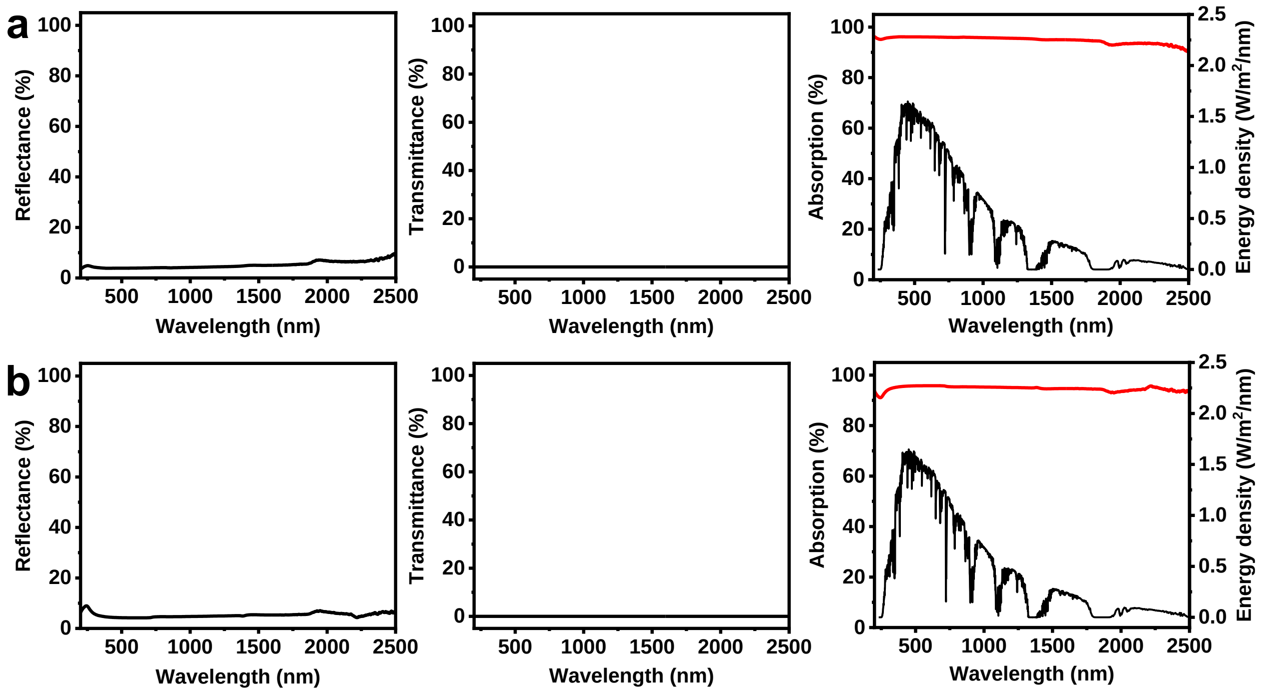

Supplementary Fig. 47 | Reflectance, transmittance, and absorption spectra and solar energy density of (**a**) biomass flash graphene from rice straw and (**b**) industrial graphene over the wavelength range of 250-2500 nm. Industrial graphene (CAS No.7782-42-5) was purchased from Aladdin® Chemicals (Shanghai, China) and was thermal reduced in hydrogen at 600°C.

Supplementary Table 15. Life-cycle inventory data for the three existing traditional graphene production technologies at the commercial scale.

Graphene (1g)	EE	COR	COT
Electricity/MJ	1.85	0.008	0.011
Graphite/g	2.29	0.89	1.19
Potassium hydroxide/g	1.12	--	--
Water (deionized)/kg	0.67	0.28	0.37
Ammonia/g	--	0.09	--
Hydrazine/g	--	1.38	--
Ethanol/g		10	--
Argon/g	--	--	3.87
Sulphuric acid aq. (96%)/g	--	3.78	5.04
Hydrogen peroxide (100%)/g	--	1.55	2.07
Sodium nitrate/g	--	0.45	0.60
Potassium permanganate/g	--	2.68	3.57
Calcium hydroxide/g	--	2.85	3.80

Data source: Cossutta et al., 2017 [1].

EE.: electrochemical exfoliation

COR.: chemical oxidation and subsequent chemical reduction

COT.: chemical oxidation and subsequent thermal reduction

Supplementary Table 16. Life cycle impacts associated with 1 g of flash graphene produced by the three traditional graphene production technologies of electrochemical exfoliation of graphite (EE), chemical oxidation and subsequent chemical reduction (COR), and chemical oxidation and subsequent thermal reduction (COT).

Item	EE	COR	COT
Climate change/g CO_2e	494	60.0	26.3
Fossil depletion/g oil-eq	151	18.4	7.69
Terrestrial acidification/g SO_2-eq	1.75	0.29	0.07
Freshwater eutrophication/g P-eq	0.09	0.02	0.01
Metal depletion/g Cu-eq	0.14	0.19	0.01
Particulate matter formation/mg PM2.5-eq	0.77	0.10	0.03
Photochemical oxidant formation/g NO_x-eq	1.34	0.15	0.05
Water depletion/dm^3	2.06	3.46	0.72

Supplementary Table 17. The construction and maintenance cost for FJH plant [a].

	300 °C	600 °C	750 °C	900 °C
Initial capital plant cost ($\times 10^4$ US$ plant^{-1})	836	2250	2250	2250
Price of feedstock (US$ Mg^{-1})	28.2	28.2	28.2	28.2
Electricity price (US$ kWh^{-1})	0.08	0.08	0.08	0.08
Device depreciation (US$ yr^{-1})	9594	9594	9594	9594
Price of gas (US$ m$^{3\ -1}$)	0.12	0.28	0.28	0.28
Price of oil (US$ Mg^{-1})	106.56	158.81	158.81	158.81

References: He et al. (2018)[2], Xia et al. (2018)[3] and Xia et al. (2023)[4].

Supplementary Table 18. Overall life-cycle inventory data to produce 1 g of flash graphene through the systems.

	Path A	Path B	Path C	Path D	Path E	Path F	Path G	Path H	Path I	Path J
**Input**										
Electricity/kWh	0.006	0.006	0.005	0.005	0.005	0.009	0.007	0.009	0.006	0.006
Quartz tube/g	0.349	0.073	0.034	0.032	0.031	0.349	0.032	0.322	-	-
Carbon black/g	0.475	0.115	0.063	-	-	0.711	-	-	-	-
Biochar/g	-	2.180	5.352	5.778	6.184	-	1.234	2.057	1.116	1.128
**Output**										
Graphene/g	1.000	1.000	1.000	1.000	1.000	1.000	1.000	1.000	1.000	1.000
Bio-gas/g	4.667	0.997	-	-	-	8.232	-	-	-	-
Bio-oil/g	3.615	0.006	0.135	0.100	0.149	4.703	0.121	0.527	0.053	0.073

Bio-oil and gas produced during pyrolysis are not included in this table.

Path A: biomass-based flash graphene production;

Path B: 300°C biochar-based flash graphene production;

Path C: 600°C biochar-based flash graphene production;

Path D: 750°C biochar-based flash graphene production;

Path E: 900°C biochar-based flash graphene production;

Path F: bamboo-derived flash graphene synthesize process;

Path G: 750°C bamboo biochar-derived flash graphene synthesize process;

Path H: 750°C rice straw biochar-derived flash graphene synthesize process;

Path I: 750°C sawdust biochar-derived flash graphene synthesize process at pilot-scale;

Path J: 750°C rice straw biochar-derived flash graphene synthesize process at pilot-scale.

Supplementary Table 19 | Comparison of purity and various application performance of low-purity and high-purity biomass FG. Industrial graphene (CAS No.7782-42-5) was purchased from Aladdin® Chemicals (Shanghai, China) and was thermal reduced in

	High-purity biomass FG	Low-purity biomass FG	Industrial graphene
Purity (%)	99.2	55.8	96.4
Dispersibility (%)	81.9	62.6	84.6
Catalytic performance (%)	93.3	63.4	99.9
Photothermal conversion (%)	92.4	94.9	94.7
Thermal conductivity (W m^{-1} K^{-1})	158	64.9	122

hydrogen at 600°C.

Supplementary references

(1) Wu, X.; Liu, Y.; Yang, H.; Shi, Z., Large-scale synthesis of high-quality graphene sheets by an improved alternating current arc-discharge method. *RSC Advances* **2016,** *6*, 93119-93124.

(2) Yousef, S.; Mohamed, A.; Tatariants, M., Mass production of graphene nanosheets by multi-roll milling technique. *Tribology International* **2018,** *121*, 54-63.

(3) Paton, K. R.; Varrla, E.; Backes, C.; Smith, R. J.; Khan, U.; O'Neill, A.; Boland, C.; Lotya, M.; Istrate, O. M.; King, P.; Higgins, T.; Barwich, S.; May, P.; Puczkarski, P.; Ahmed, I.; Moebius, M.; Pettersson, H.; Long, E.; Coelho, J.; O'Brien, S. E.; McGuire, E. K.; Sanchez, B. M.; Duesberg, G. S.; McEvoy, N.; Pennycook, T. J.; Downing, C.; Crossley, A.; Nicolosi, V.; Coleman, J. N., Scalable production of large quantities of defect-free few-layer graphene by shear exfoliation in liquids. *Nat. Mater.* **2014,** *13*, 624-630.

(4) Knieke, C.; Berger, A.; Voigt, M.; Taylor, R. N. K.; Röhrl, J.; Peukert, W., Scalable production of graphene sheets by mechanical delamination. *Carbon* **2010,** *48*, 3196-3204.

(5) Van Grinsven, H. J. M.; Holland, M.; Jacobsen, B. H.; Klimont, Z.; Sutton, M. a.; Jaap Willems, W., Costs and Benefits of Nitrogen for Europe and Implications for Mitigation. *Environ. Sci. Technol.* **2013,** *47*, 3571-3579.

(6) Bayazit, M. K.; Xiong, L.; Jiang, C.; Moniz, S. J. A.; White, E.; Shaffer, M. S. P.; Tang, J., Defect-Free Single-Layer Graphene by 10 s Microwave Solid Exfoliation and Its Application for Catalytic Water Splitting. *ACS Appl. Mater. Interfaces* **2021,** *13*, 28600-28609.

(7) Herron, C. R.; Coleman, K. S.; Edwards, R. S.; Mendis, B. G., Simple and scalable route for the 'bottom-up' synthesis of few-layer graphene platelets and thin films. *J. Mater. Chem.* **2011,** *21*, 3378-3383.

(8) Gao, H.; Zhu, K.; Hu, G.; Xue, C., Large-scale graphene production by ultrasound-assisted exfoliation

of natural graphite in supercritical CO2/H2O medium. *Chem. Eng. J.* **2017,** *308*, 872-879.

(9) Geng, X.; Guo, Y.; Li, D.; Li, W.; Zhu, C.; Wei, X.; Chen, M.; Gao, S.; Qiu, S.; Gong, Y.; Wu, L.; Long, M.; Sun, M.; Pan, G.; Liu, L., Interlayer catalytic exfoliation realizing scalable production of large-size pristine few-layer graphene. *Sci. Rep.* **2013,** *3*, 1134.

(10) Feng, H.; Cheng, R.; Zhao, X.; Duan, X.; Li, J., A low-temperature method to produce highly reduced graphene oxide. *Nat. Commun.* **2013,** *4*, 1539.

(11) Chakrabarti, M. H.; Manan, N. S. A.; Brandon, N. P.; Maher, R. C.; Mjalli, F. S.; AlNashef, I. M.; Hajimolana, S. A.; Hashim, M. A.; Hussain, M. A.; Nir, D., One-pot electrochemical gram-scale synthesis of graphene using deep eutectic solvents and acetonitrile. *Chem. Eng. J.* **2015,** *274*, 213-223.

(12) Lu, W.; Liu, S.; Qin, X.; Wang, L.; Tian, J.; Luo, Y.; Asiri, A. M.; Al-Youbi, A. O.; Sun, X., High-yield, large-scale production of few-layer graphene flakes within seconds: using chlorosulfonic acid and H2O2 as exfoliating agents. *J. Mater. Chem.* **2012,** *22*, 8775-8777.

(13) Wang, J.; Manga, K. K.; Bao, Q.; Loh, K. P., High-Yield Synthesis of Few-Layer Graphene Flakes through Electrochemical Expansion of Graphite in Propylene Carbonate Electrolyte. *J. Am. Chem. Soc.* **2011,** *133*, 8888-8891.

(14) Zhou, X.; Liu, Z., A scalable, solution-phase processing route to graphene oxide and graphene ultralarge sheets. *Chem. Commun.* **2010,** *46*, 2611-2613.

(15) Moon, I. K.; Lee, J.; Ruoff, R. S.; Lee, H., Reduced graphene oxide by chemical graphitization. *Nat. Commun.* **2010,** *1*, 73.

(16) Choi, E.-Y.; Choi, W. S.; Lee, Y. B.; Noh, Y.-Y., Production of graphene by exfoliation of graphite in a volatile organic solvent. *Nanotechnology* **2011,** *22*, 365601.

(17) Shang, N. G.; Papakonstantinou, P.; Sharma, S.; Lubarsky, G.; Li, M.; McNeill, D. W.; Quinn, A. J.; Zhou, W.; Blackley, R., Controllable selective exfoliation of high-quality graphene nanosheets and nanodots by ionic liquid assisted grinding. *Chem. Commun.* **2012,** *48*, 1877-1879.

(18) Oh, S. Y.; Kim, S. H.; Chi, Y. S.; Kang, T. J., Fabrication of oxide-free graphene suspension and transparent thin films using amide solvent and thermal treatment. *Appl. Surf. Sci.* **2012,** *258*, 8837-8844.

(19) Cossutta, M.; McKechnie, J.; Pickering, S. J., A comparative LCA of different graphene production routes. *Green Chemistry* **2017,** *19*, 5874-5884.

(20) He, X.; Liu, Z.; Niu, W.; Yang, L.; Zhou, T.; Qin, D.; Niu, Z.; Yuan, Q., Effects of pyrolysis temperature on the physicochemical properties of gas and biochar obtained from pyrolysis of crop residues. *Energy* **2018,** *143*, 746-756.
