# Supplementary material for: Continuous and low-carbon production of biomass flash graphene
Source: Nat Commun. 2024 Apr 15;15:3218. doi: 10.1038/s41467-024-47603-y (PMC11018853; doi:10.1038/s41467-024-47603-y)
Supplement: Supplementary file 3 — Description of Additional Supplementary Files [file 41467_2024_47603_MOESM3_ESM.pdf]

Description of Additional Supplementary Files

File Name: Supplementary Data 1
Description: The data that supports the LCA of the study.

File Name: Supplementary Movie 1
Description: Continuous flash graphene production under 16x fastforward play.

File Name: Supplementary Movie 2
Description: Two consecutive flash graphene production cycles.
